# Synthetic Lethality between Gene Defects Affecting a Single Non-essential Molecular Pathway with Reversible Steps

**DOI:** 10.1371/journal.pcbi.1003016

**Published:** 2013-04-04

**Authors:** Andrei Zinovyev, Inna Kuperstein, Emmanuel Barillot, Wolf-Dietrich Heyer

**Affiliations:** 1 Institut Curie, Paris, France; 2 INSERM, U900, Paris, France; 3 Mines ParisTech, Fontainebleau, France; 4 Departments of Microbiology & Molecular Genetics and of Molecular & Cellular Biology, University of California, Davis, Davis, California, United States of America; University of Toronto and Mount Sinai Hospital, Canada

## Abstract

Systematic analysis of synthetic lethality (SL) constitutes a critical tool for systems biology to decipher molecular pathways. The most accepted mechanistic explanation of SL is that the two genes function in parallel, mutually compensatory pathways, known as between-pathway SL. However, recent genome-wide analyses in yeast identified a significant number of within-pathway negative genetic interactions. The molecular mechanisms leading to within-pathway SL are not fully understood. Here, we propose a novel mechanism leading to within-pathway SL involving two genes functioning in a single non-essential pathway. This type of SL termed within-reversible-pathway SL involves reversible pathway steps, catalyzed by different enzymes in the forward and backward directions, and kinetic trapping of a potentially toxic intermediate. Experimental data with recombinational DNA repair genes validate the concept. Mathematical modeling recapitulates the possibility of kinetic trapping and revealed the potential contributions of synthetic, dosage-lethal interactions in such a genetic system as well as the possibility of within-pathway positive masking interactions. Analysis of yeast gene interaction and pathway data suggests broad applicability of this novel concept. These observations extend the canonical interpretation of synthetic-lethal or synthetic-sick interactions with direct implications to reconstruct molecular pathways and improve therapeutic approaches to diseases such as cancer.

## Author Summary

Organizing gene functions into molecular pathways is a major challenge in biology. The observation that two viable gene mutations become lethal when combined as a double mutant has been developed into a major genetic tool called synthetic lethality. The classic interpretation of synthetic lethality stipulates that the two mutations identify genes that work in parallel, mutually compensatory pathways that together perform an essential function. However, a significant number of negative interactions are caused by defects affecting a single molecular pathway. Here, we recapitulate by mathematical modeling recent experimental data that demonstrate synthetic lethality between mutations in genes acting in a single, non-essential molecular pathway. We propose a novel mechanism involving reversible pathways steps and trapping of an intermediate. The modeling also predicts that overexpression of certain genes functioning in reversible pathways will lead to synthetic lethality with gene defects in the same pathway. Our results significantly broaden the interpretation of synthetic lethal and synthetic dosage effects, which fundamentally impacts the assignment of genes to pathways. The concept of synthetic lethality has been applied to cancer therapy, and our modeling results suggest new approaches to how to target a single pathway to induce synthetic lethality in cancer cells.

## Introduction

Synthetic interactions between two mutations in different genes were first revealed in *Drosophila* by Dobzhansky in the 1940s [1]. Synthetic lethality (SL) describes that two viable single gene mutations lead to lethality (synthetic-lethal) or severely impair growth (synthetic-sick) when combined as a double mutant. This concept was implemented as a powerful research tool for molecular pathway analysis in yeast [2]–[5]. Further refinement introduced more quantitative measures of genetic epistasis [6] and lethality induced by gene overexpression in a mutant background (synthetic dosage-lethality [7]). A genetic interaction is negative or aggravating, when the combined effect of two gene defects is more severe than it is expected from a simple multiplicative model. In a positive or alleviating interaction the effect is less severe than expected. These approaches and measures are now increasingly used in mammalian cells exploiting RNA-mediated gene knockdown technologies [8], [9].

Following a proposal by Hartwell and colleagues [10], SL has been utilized as a therapeutic approach in cancer treatment employing a combination of genetic ablation (loss of tumor suppressor) and chemical inhibition. The first paradigm was set in BRCA1/2-deficient tumor cells, which are synthetic-lethal with inhibition of PolyADP-Ribose Polymerase (PARP) [11]–[13]. Small molecule PARP inhibitors are currently being evaluated in clinical trials in BRCA1- and BRCA2-deficient cancers (*e.g.* [14]).

**Figure 1:**
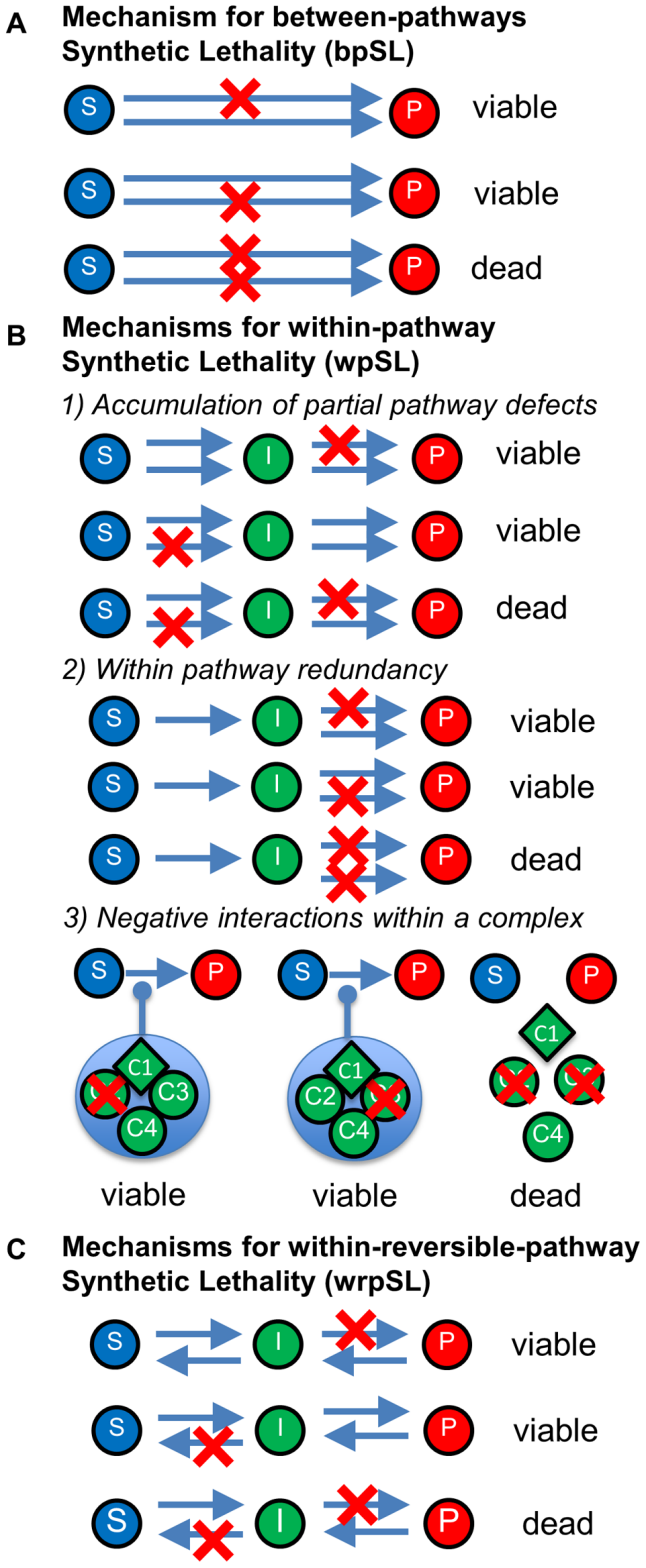
Schematic representation of mechanisms of (A) between-pathways Synthetic Lethality, (B) within-pathway Synthetic Lethality, and (C) within-reversible-pathway Synthetic Lethality. S: substrate, I: intermediate, P: product, C1–4: components of a protein complex. Red crosses define single mutations. For details see text.

The canonical interpretation of SL stipulates two mutually compensatory, parallel pathways capable of performing the same essential function [2]–[4]. Thus, disrupting a single pathway is viable, while disrupting both pathways is lethal. This concept of between-pathway synthetic lethality (bpSL) (Figure 1A) led to the creation of computational approaches aiming at reconstructing interaction networks from pair-wise gene deletions or siRNA-induced gene knock-down screens in yeast and mammals [15]–[17]. However, recent genome-wide genetic interaction data revealed multiple negative interactions between mutations affecting the same molecular pathway or complex [3], [5],[16],[18]–[21]. For example, it was estimated that ∼9% [19] and in another study 14% [17] of all negative genetic interaction clusters belong to the same biological pathway. Several mechanistic models were suggested to explain within-pathway SL (wpSL) [6], [16], [20], [22]. The deletion of a gene might lead only to a partial degradation of an essential pathway which might be tolerable, whereas the double mutation leads to complete pathway degradation and lethality (Figure 1B1). This is especially relevant for the interpretation of siRNA-based screens where the efficiency of a particular gene knock down is uncertain. A second possible mechanism suggests that steps in an essential pathway are internally redundant (Figure 1B2). Lastly, two mutations may cumulatively degrade an essential protein complex, whereas they are individually viable (Figure 1B3). This mechanism is consistent with the observations that molecular complexes are frequently characterized by the dominance of negative over positive genetic interactions between their components [18]. wpSL interactions between defects in components of a single protein complex are highly enriched for complexes with an essential component [16], [22]. It was estimated that the contribution from within-complex interactions to the total number of within-pathway negative interactions does not exceed 7% [19]. Common to these mechanistic explanations of wpSL is that they involve either an essential pathway or an essential protein complex.

Here, we highlight a novel scenario of wpSL involving two components of a non-essential pathway. The view of molecular pathways as unidirectional, linear reaction cascades is too simplistic. Pathway steps can be reversible which leads to forward and backward propagation of molecular events along the pathway increasing robustness and fidelity of the overall process [23]–[28]. Affecting both forward and reverse steps of the pathway by abrogating the corresponding enzymes creates scenarios in which the pathway flow can be trapped in an intermediate state that may be toxic to the cell or deprive the cell of a limiting resource (Figure 1C). This can create a genetic scenario we define as within-reversible-pathway synthetic lethality (wrpSL), which is the subject of this study.

Here, we study bpSL and wrpSL scenarios using mathematical modeling to better understand the system properties of these genetic relationships. We present a simplified model of the pathway applicable for its formal analytical study and performed *in silico* simulations for bpSL and wrpSL as well as synthetic dosage effects. Our main experimentally confirmed examples of wrpSL are in the homologous recombination DNA repair pathway. Homologous recombination (HR) is an important mechanism to maintain genome integrity [29] (Section S1 and Figure S1 in for more discussion). Analysis of yeast gene interaction and pathway data suggests broad applicability of this novel concept.

## Results

### Homologous recombination and other DNA repair pathways are a hotspot for negative genetic interactions

**Figure 2:**
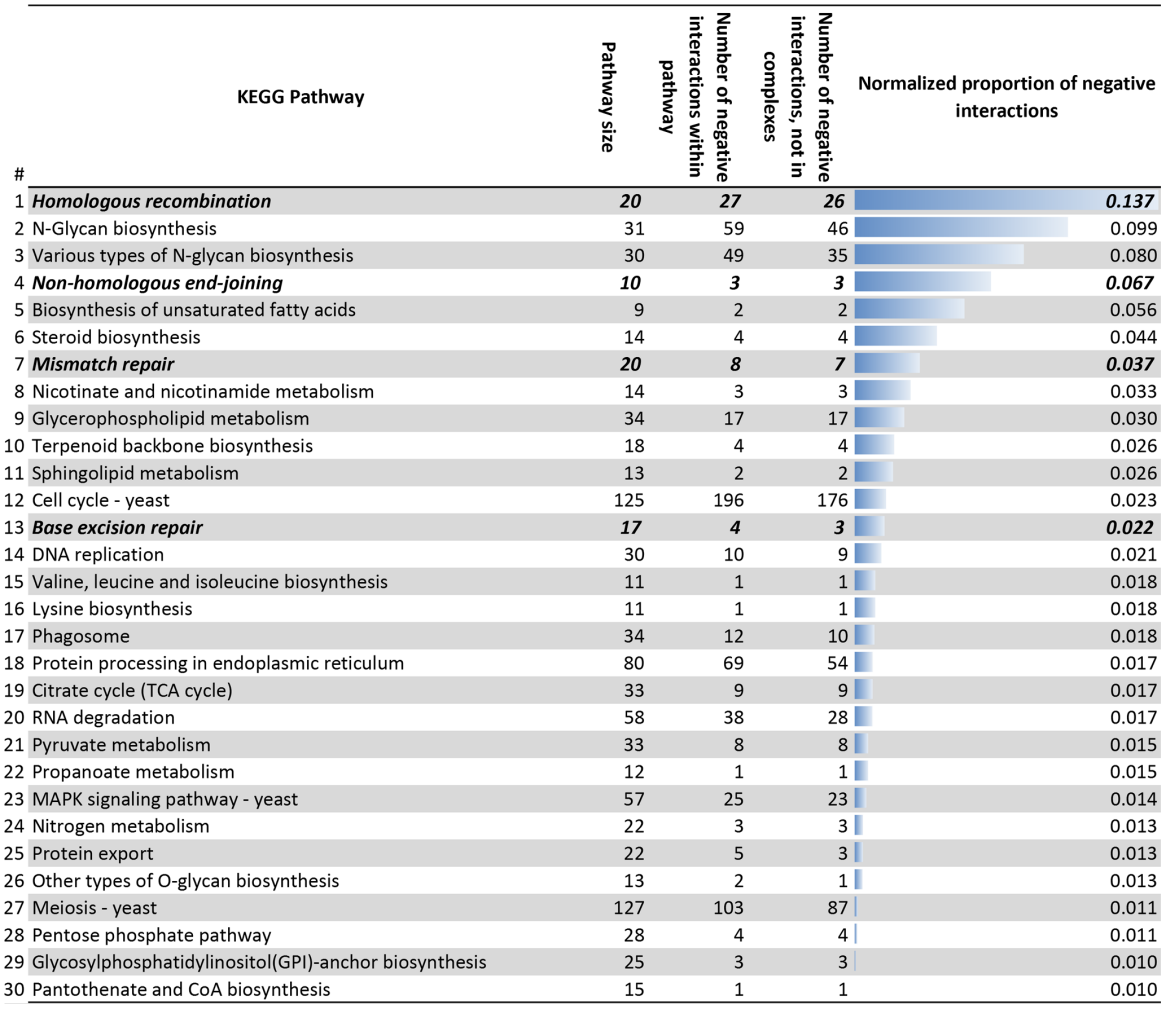
Normalized proportion of negative genetic interactions within one KEGG pathway. The proportion is related to the number of all possible pairwise interactions *S*(*S*−1)/2, where *S* is the size of the KEGG pathway. Negative interactions between components of a molecular complex were excluded (compare the fourth and the fifth columns). We counted the normalized number of negative genetic interactions within one pathway using recent data on the genome-wide screening of genetic interactions in yeast from [5], using the most stringent filter on the epistasis measure ε. Definitions of yeast signaling and metabolic pathways were taken from KEGG database [30]. Only KEGG pathways with a normalized proportion of ≥1% are shown. DNA repair KEGG pathways are highlighted in bold.

In order to assess the importance of within-pathway negative interactions we ranked all pathways from the KEGG database [30] according to their normalized proportion of negative interactions [5] within each KEGG pathway (Figure 2). This analysis confirms the previous conclusion [19] that only a minority of within-pathway negative interactions can be explained by negative interactions within a complex (Figure 1B3). In our analysis only 12% of all negative interactions were of this type (compared to 7% in [19]). Interestingly, HR ranks at the top with 27 within-pathway negative interactions between 20 KEGG pathway components (Figure 2), of which only a single one affects components of the same protein complex.

### Reversibility of the pathway steps in homologous recombination pathway

Recent studies show that individual reaction steps in HR are reversible [23]–[25] (Figure S1 in Text S1). The Rad51-ssDNA filament is a key intermediate in HR, as it performs the signature reactions of homology search and DNA strand invasion. The formation of this filament is catalyzed by specific co-factors (see Section S1 and Figure S1 in Text S1). The helicase Srs2 specifically targets the Rad51-ssDNA filament for disruption to reverse filament formation [31], [32], [33]. The reversibility of the Rad51-ssDNA filament sets a new paradigm and draws attention to additional reversible steps and their mechanisms in HR, other DNA repair processes, and unrelated molecular pathways.

### Simplest abstract toy model of DNA repair pathway with reversible steps and a toxic intermediate

**Figure 3:**
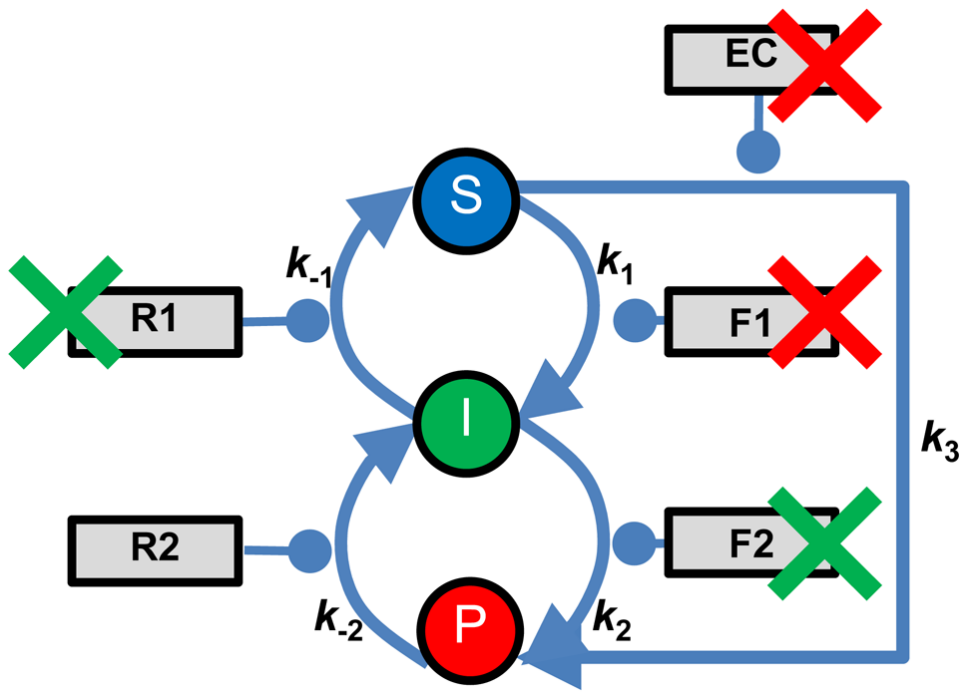
Abstract representation of the recombinational DNA repair pathway. The scheme represents a simplified version of the pathway depicted in Figure S1 in Text S1 using the same abbreviations. Dynamic states **S**, **I**, **P** represent DNA damage substrate (**S**), toxic intermediate (**I**), and the product of repair (**P**). **F1** (*e.g.* Rad51), **F2** (*e.g.* Rad54), **R1** (*e.g.* Srs2), **R2** (*e.g.* Mph1) are enzymes in the main pathway, and **F3-EC** represent enzymes in the compensatory pathway. *k*s signify the kinetic rates of the model state transition steps. The two types of synthetic lethality (SL) are indicated: Red Xs – classic scenario of between-pathway SL (*e.g. BRCA1* or *BRCA2* mutant and PARP inhibition); Green Xs – within-reversible-pathway SL between two mutations in genes acting in a single non-essential pathway leading to the accumulation of a toxic intermediate (*e.g. srs2 rad54* double mutant). For more discussion see also Section S1 in Text S1.

**Figure 4:**
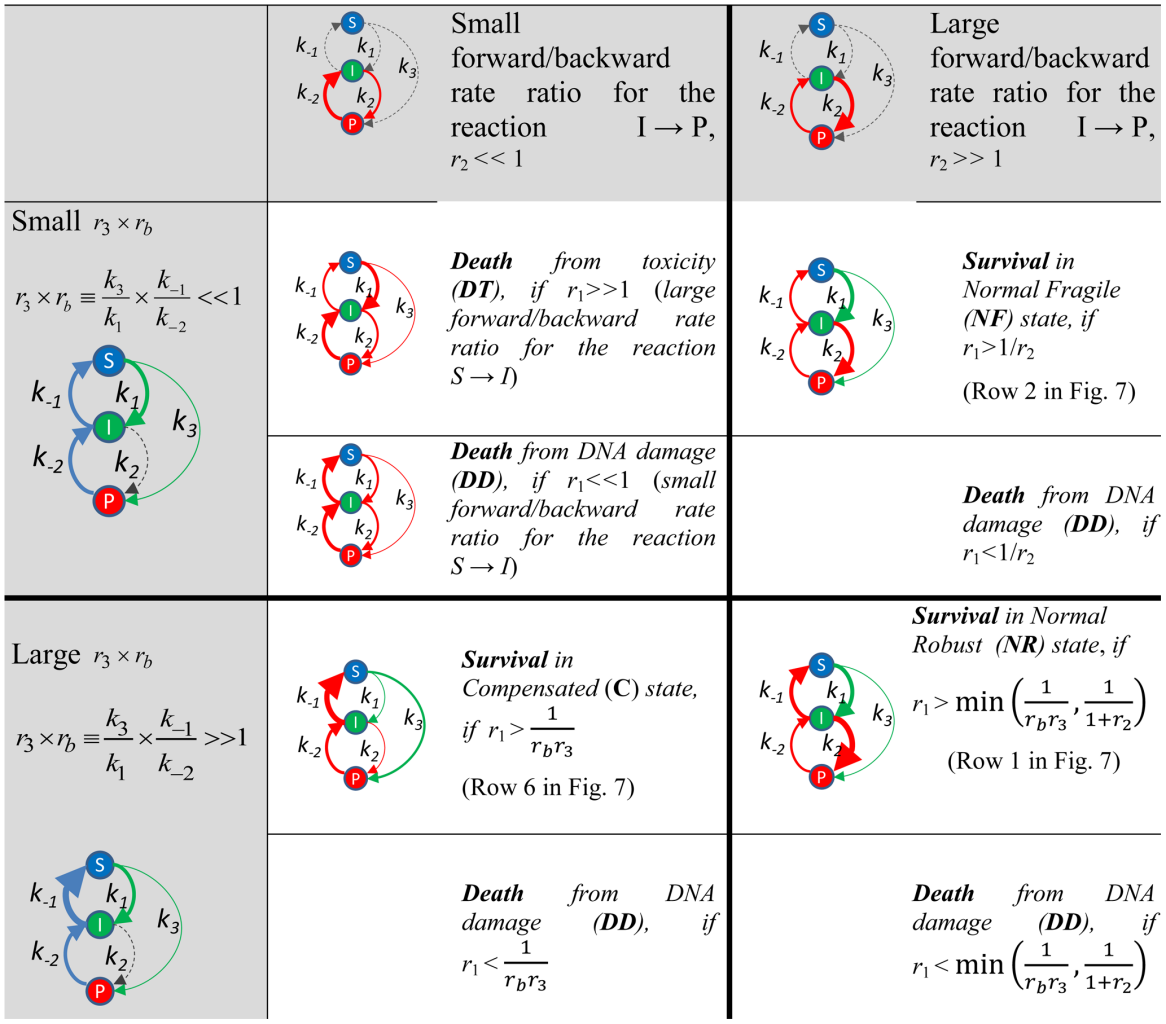
Pathway steady states for various combinations of parameters in the mathematical model of DNA repair with reversible steps and a toxic intermediate. The classification of pathway steady states depends on the values of three control parameters, representing the ratios of some kinetic rates of the model (for more details see Section S2A in Text S1): 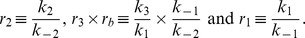 The diagrams visualize the values of the control parameters where thickness of the solid arrows shows the relative value of the corresponding kinetic rate and the dashed arrows represent kinetic rates which values are irrelevant for a given scenario. Color coding shows partial orderings of the parameters important for a given pathway state (thickness of edges of the same color should be compared but not between color.

**Figure 5:**
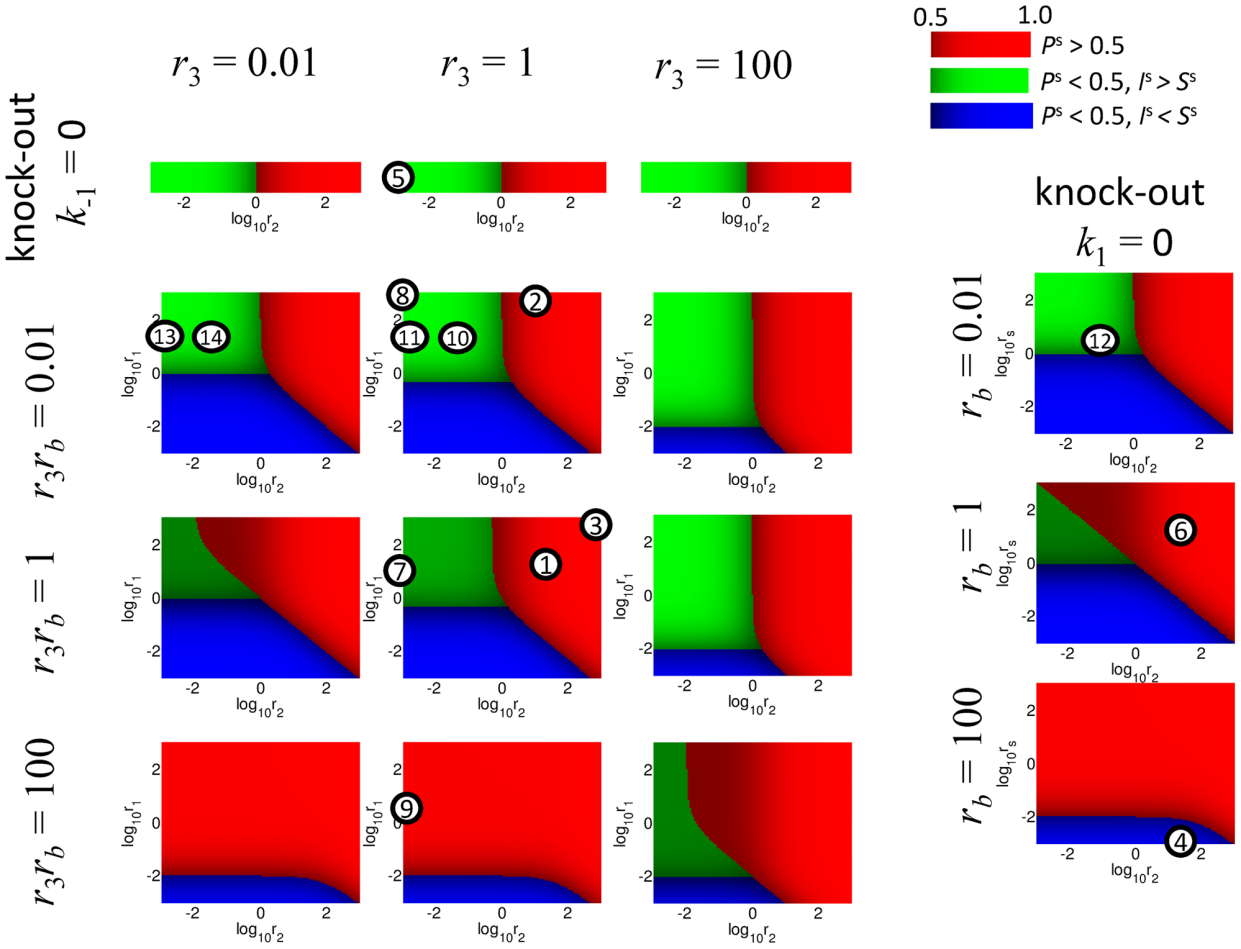
Visualization of the steady states for the toy model in dependence of various parameters. The phase diagrams (*r*_2_×*r*_1_ plane) show the qualitative behavior for small, intermediate and large values of the model parameters. Red color corresponds to the state for which *P^s^*>0.5 (DNA is repaired with probability >50%). If *P^s^*<0.5 then the color is chosen as green if the probability of trapping in the intermediate state (*I*) is bigger than the propability of the initial unrepaired state (*S*), and as blue in the opposite case. The case *k*_1_ = 0 (F1↓) is represented separately on the right. In this case, another parameter 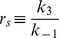 is used instead of *r*_1_ for the phase plane. The case *k−*_1_ = 0 (R1↓) is treated separately on the top and only the *r*_2_ value is varied. The 14 model simulations listed in Figure 6 are shown by the circled numbers in the position of the chosen parameters.

We derived the simplest linear mathematical model of a main DNA repair pathway with reversible steps and a toxic intermediate, and a compensatory pathway, which can recapitulate bpSL and wrpSL (Figure 3). Each state transition is catalyzed by an abstract enzyme, which may correspond to several biological entities (compare Figure S1 in Text S1 with Figure 3). In wrpSL trapping of toxic intermediate I is caused by defects in the first backward reaction (I→S, R1, *k*_−1_) and the second forward reaction (I→P, F2, *k*_2_). The reversibility of the second step (reaction P→I, R1) is not essential for wrpSL to occur, but might be important for quantitative pathway characteristics. Introduction of a final irreversible step (Figure S2 in Text S1) would result in a kinetic proofreading mechanism [34] (see Figures S2, S3 in Text S1 and discussion there). Such a mechanism increases the robustness of DNA repair, as it avoids a futile P↔I cycle. However, in this simplest model we eliminated the final irreversible step to allow us analyzing the most essential features of wrpSL (Section S2 in Text S1). Figure 4 explores conditions for various cellular fates (Normal Robust, ***NR***: no single knockout leads to lethality (Figure S4 in Text S1); Normal Fragile, ***NF***: single knockout can lead to lethality (Figure S4 in Text S1); Compensated, ***C***: repair is performed by compensatory pathway; death due to DNA Damage, ***DD***: steady state probability of DNA damage >50%; and Death due to Toxic intermediates, ***DT***: steady state probability of toxic intermediate >50%). Figure 5 visualizes parametric conditions (see Section S2B in Text S1 for discussion).

### Examples of numerical simulations of the toy model

**Figure 6:**
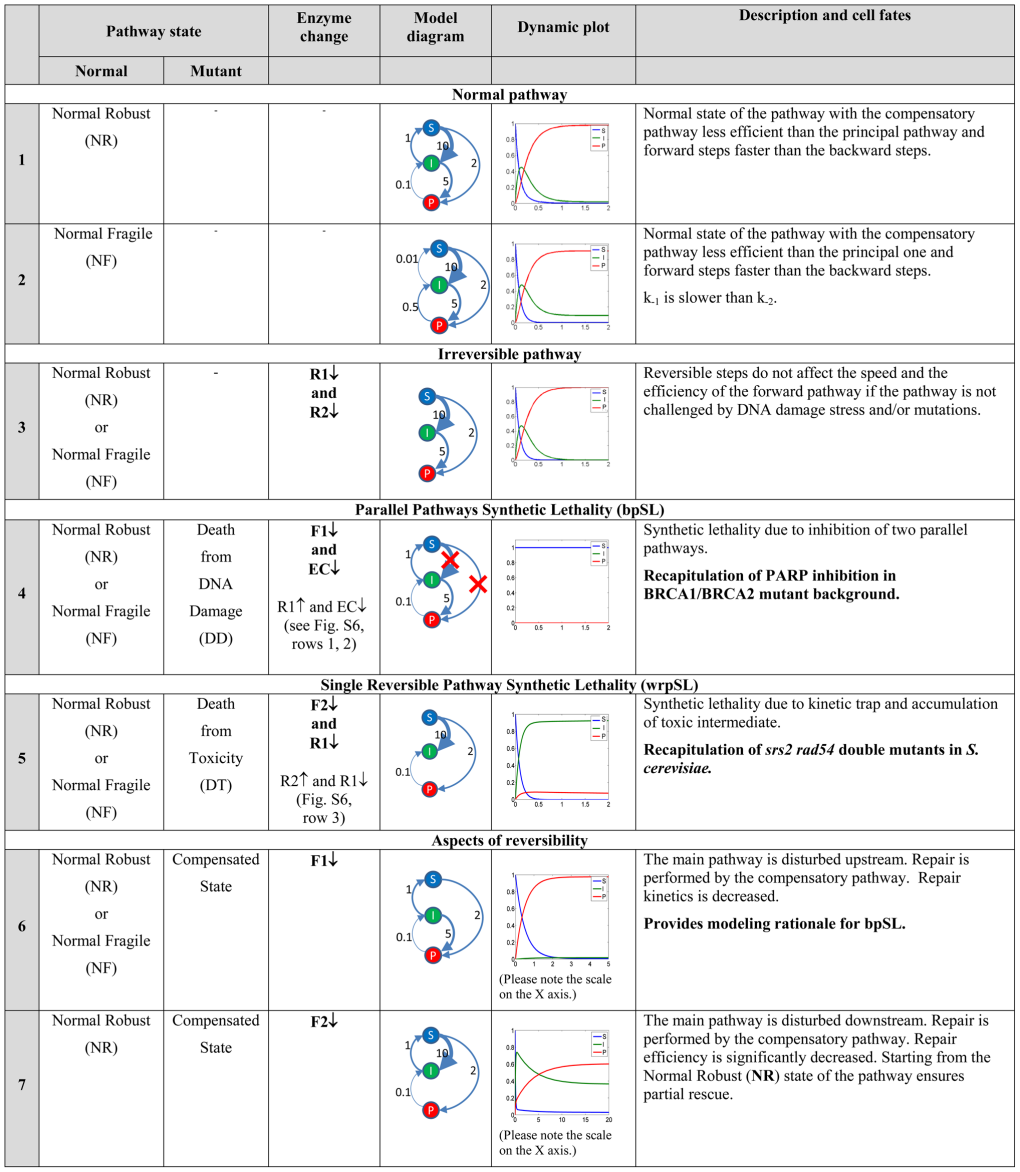
Modeling possible scenarios of single and double synthetic lethal mutations. Pathway steady states depicted as **Normal states** (**N**) as Normal Robust (**NR**) and Normal Fragile (**NF**); **Mutant states** (**M**) as compensated state dependent on compensatory pathway EC(**C**); death from DNA damage (**DD**) and death from toxicity (**DT**). **Dynamic plots** show prediction of model for evolution of substrate (**S**), intermediate (**I**) and product (**P**) amounts over time corresponding to the choice of kinetic parameters shown on the **Model diagram**. (**X-axis**)-time, (**Y-axis**)-substances level. **F1**, **F2**, **R1**, **R2**, and **EC** refer to the enzymes catalyzing the two forward and two backward reactions as well as the compensatory pathway, respectively (see Figure 3). (↓)-complete knock-down or mutational loss of function; (↑)-over-expression. A deletion mutant was simulated by setting the corresponding kinetic rate constant to zero. An overexpression mutant was simulated by setting the corresponding kinetic rate constant sufficiently high to have a qualitative effect onto the steady state or the dynamics in the simulations. Some double mutants are not shown due to their triviality (such as F1↓F2↓) or difficulties with interpretation (such as F1↓R1↓). For more modeling scenarios see Figure S5 in Text S1.

Using analytical study and numerical simulations with some characteristic choices of kinetic rate values, we explored the dynamical behaviors of the simplest model (see Figure 6). Here, we discuss the qualitative results and interpretations, while the more formal derivation of these statements is found in Section S3 in Text S1. To illustrate the static and dynamic properties of the toy model, we selected two typical positions (Figure 5 #1, #2) corresponding to ***NR*** and ***NF*** pathway states, respectively. From these “normal” conditions we simulated a number of single and double mutant/overexpression conditions as shown in Figure 5 (see also Figure S4 in Text S1).

#### Normal pathway vs. irreversible pathway: reversibility does not affect efficiency, but is essential for pathway robustness

**Figure 7:**
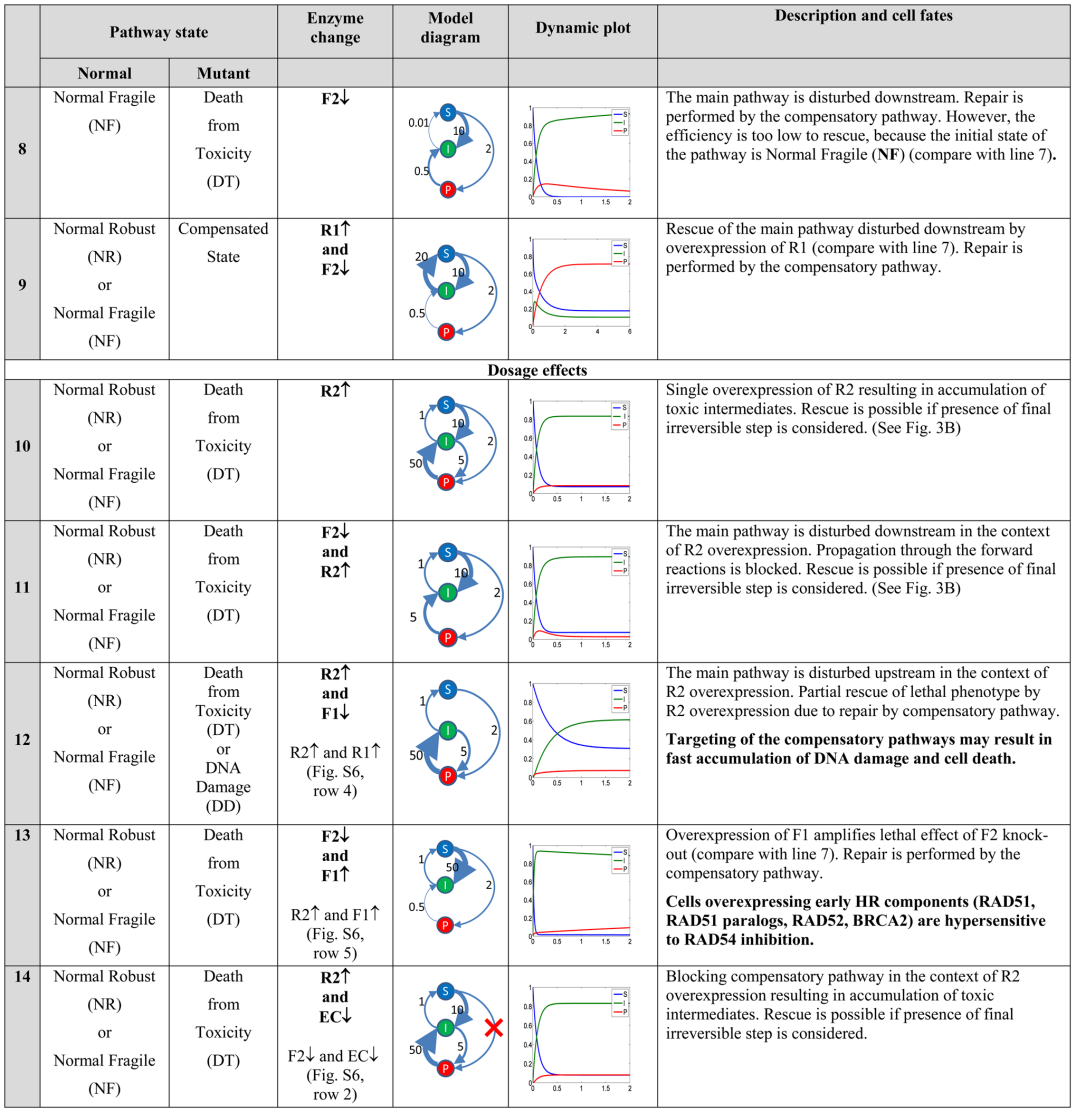
Modeling possible scenarios of single and double synthetic lethal mutations (continued). Pathway steady states depicted as **Normal states** (**N**) as Normal Robust (**NR**) and Normal Fragile (**NF**); **Mutant states** (**M**) as compensated state dependent on compensatory pathway EC(**C**); death from DNA damage (**DD**) and death from toxicity (**DT**). **Dynamic plots** show prediction of model for evolution of substrate (**S**), intermediate (**I**) and product (**P**) amounts over time corresponding to the choice of kinetic parameters shown on the **Model diagram**. (**X-axis**)-time, (**Y-axis**)-substances level. **F1**, **F2**, **R1**, **R2**, and **EC** refer to the enzymes catalyzing the two forward and two backward reactions as well as the compensatory pathway, respectively (see Figure 3). (↓)-complete knock-down or mutational loss of function; (↑)-over-expression. A deletion mutant was simulated by setting the corresponding kinetic rate constant to zero. An overexpression mutant was simulated by setting the corresponding kinetic rate constant sufficiently high to have a qualitative effect onto the steady state or the dynamics in the simulations. Some double mutants are not shown due to their triviality (such as F1↓F2↓) or difficulties with interpretation (such as F1↓R1↓). For more modeling scenarios see Figure S5 in Text S1.

We assume that in the ***NR*** and ***NF*** states under normal conditions forward steps of the pathways are faster than the backward steps. In this case the presence of reversible steps does not significantly affect the numerical solutions of the model equations, *i.e.* the efficiency of DNA repair (Figure 5 cases 1–3; Figure 6, rows 1–3; Figure S4 in Text S1). However, the presence of reversible steps is important from the robustness point of view (Figure 5 cases 3, 7; Figure 6, rows 3, 7; Figure S4 in Text S1). The gain in robustness through backward reactions is also illustrated by the fact that the difference between the ***NR*** and ***NF*** states is entirely defined by the ratio of the two backward reactions (*r_b_*). The model recapitulates the fact that single enzyme deficiencies are not capable to completely block the overall DNA repair network. The F2 knock-out in ***NF*** is the only exception. When the first backward reaction is relatively slow 
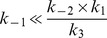
, the compensatory pathway cannot rescue the lethal phenotype (Figure 5 case 8; Figure 7, row 8; Figure S4 in Text S1). This underlines the importance of efficient backward reactions.

#### Between-pathway Synthetic Lethality (bpSL): Disruption of main and compensatory pathways

The model recapitulates canonical bpSL by putting two kinetic rates to zero simultaneously: *k*_1_ = *k*_3_ = 0 which causes cell death from accumulation of DNA damage (***DD***) (Figure 5 case 4; Figure 6, row 4; Figure S4 in Text S1). Alternatively, in the case of *k*_2_ = *k*_3_ = 0 the pathway state will be ***DT***, resulting in cell death from accumulation of toxic intermediates.

#### Within-reversible-pathway Synthetic Lethality (wrpSL): Trapping a toxic intermediate

The wrpSL case is modeled by putting 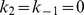. As it can be seen (Figure 5 case 5; Figure 6, row 5; Figure S4 in Text S1), this creates a kinetic trap in the toxic intermediate state *I* (***DT***). The model recapitulates the situation observed in the *srs2 rad54* double mutant [23], [35], [36]. Modeling predicts that mammalian cells deficient in RAD54 or its close paralog RAD54B should also be sensitive to inhibition of enzymes, such as FBH1, FANCJ, or RECQ5 [37]–[39] (Figure 5 cases 5, 8; Figure 6, row 5; Figure 7, row 8; Figure S4 in Text S1), that are postulated or shown to dissociate the RAD51-ssDNA filament (Figures S1, S2 in Text S1).

#### Aspects of reversibility

Blocking the pathway upstream by eliminating the F1 reaction in the normal pathway states (***NR*** and ***NF***) does not lead to cell death. However, this affects the kinetics of the process, because repair is carried out by the compensatory pathway (state C), which is slower than the principal one (Figure 5 case 6; Figure 6, row 6; Figure S4 in Text S1). Therefore, the time of repair increases and the cell fate depends on the efficiency of the compensatory pathway, which might be cell type-specific. The situation may result in cell death if the kinetics of the process are too slow or the efficiency of DNA damage repair by the compensatory pathway is below an essential threshold. This scenario may represent cancer cells with certain DNA repair defects, which can be compensated by alternative pathways. In such cases, therapeutic targeting of the compensatory pathways may result in fast accumulation of DNA damage, selectively killing the cancer cells, representing the classic bpSL concept.

When the pathway is blocked downstream at F2 (Figure 5 case 7; Figure 6, row 7; Figure S4 in Text S1) the time of repair significantly increases, as the pathway is only partially compensated. This scenario can be rescued by reducing *k*_1_ or increasing *k*_−1_ (state **C**). The scenario recapitulates RAD54-deficient cells, where sub-lethal levels of toxic intermediates and unrepaired DNA increase genetic instability [36], [40]. This last scenario of a downstream block (F2 knockout: Figure 5 case 7; Figure 6, row 7; Figure S4 in Text S1) can be rescued by overexpression of R1 (Figure 5 case 9; Figure 7, row 9; Figure S4 in Text S1). The modeling predicts that this will accelerate the backward reactions, favor the compensatory route and increase repair of damaged DNA by the compensatory pathway (state **C**). The modeling also highlights the benefit of reversibility. In Figure 7 (row 8), a decrease in reversibility (10-fold reduction in R1) leads to significantly less repaired product in mutants that block the downstream step (F2). Complete inhibition of the R1 step leads to wrpSL in cells lacking F2, such as RAD54B-deficient cells (Figure 5 case 8; Figure 7, row 8; Figure S4 in Text S1, see above). The modeling suggests that cells deficient in the downstream forward reaction (F2, *e.g.* RAD54B^−/−^) are potentially under selective pressure to strengthen the upstream backward reaction (R1, *e.g.* overexpression of FBH1, FANCJ, or RECQ5).

#### Activating mutations or overexpression can create within-reversible-pathway-synthetic dosage lethality

The computational model allows making predictions in less intuitive cases of model perturbations. The lethal phenotype by R2 overexpression (Figure 5 case 10; Figure 7, row 10; Figure S4 in Text S1) can be partially rescued by eliminating F1 (Figure 5 case 12; Figure 7, row 12; Figure S4 in Text S1), as repair is carried out by the compensatory pathway (state **C**). The modeling illustrates how a defect in an early HR gene such as *RAD51*, *BRCA2*, *RAD52*, or the *RAD51* paralogs would provide a selective advantage in cells overexpressing enzymes of the R2 backward reaction (yeast Mph1, human RTEL1 or FANCM).

F1 overexpression amplifies the effect of an F2 mutation, creating synthetic-lethal dosage and leading to wrpSL due to the accumulation of toxic intermediates (state ***DT***) (Figure 5 case 13; Figure 7, row 13; Figure S4 in Text S1). This suggests that inhibition of F2 (for example RAD54/RAD54B) would be particularly effective to sensitize cancer cells overexpressing F1 components (*e.g.* RAD51 paralogs, RAD51, RAD52, BRAC2 [41]–[45]), a non-intuitive insight derived from the mathematical modeling.

Blocking the compensatory pathway and overexpressing R2 also creates synthetic lethal dosage interaction, causing accumulation of toxic intermediates due to the increase in backward reactions kinetics (state ***DT***) (Figure 5 case 14; Figure 7, row 14; Figure S4 in Text S1). However, if the final irreversible step of DNA repair will be taken into consideration (P→I2a/b→EP, Figure 3), slow conversion of all favorable DNA configurations from the futile I↔P cycle into the completely repaired DNA state will rescue cells (similar to scenario depicted in Figure 7, row 10). This example is interesting, because it demonstrates that interference with compensatory pathways by drugs might have a therapeutic importance if the backward steps of the main pathway are considered. Finally, the computational model predicts that a similar trap in the intermediate state can be created by eliminating R1 and overexpressing R2 (Figure 5 case 3; Figure S4 in Text S1; Figure S5, row 3 in Text S1).

## Discussion

Synthetic lethality/sickness and synthetic dosage lethality are important genetic tools to assign individual gene functions into molecular pathways [2]–[4], [7]–[9]. The canonical interpretation for two mutants found to be synthetically lethal or sick stipulates that the encoded gene products function in different parallel pathways that can mutually compensate (bpSL) [2]–[4], [7]–[9], [15]–[17]. However, computational analysis of genetic interaction data combined with protein interaction data revealed multiple negative interactions between mutations affecting functions in the same molecular pathway or complex (wpSL) [3], [5], [16], [18]–[21]. Several mechanisms of wpSL have been proposed (Figure 1B), and they all involve either essential pathways or essential protein complexes. In extension of this fundamental concept of wpSL, there are several cases of SL between mutants encoding proteins acting in HR, a pathway that is not essential in yeast [35], [46]–[48]. We term this novel genetic interaction within-reversible-pathway Synthetic Lethality (wrpSL; Figure 1C) and provide a novel mechanistic explanation for wpSL, which can create SL within a non-essential pathway or between hypomorphic mutations in an essential pathway that is different from a model invoking sequential pathway degradation by accumulation of partial defects of successive steps (Figure 1B1).

Here, we explore by mathematical modeling the system properties of wrpSL. The modeling must make assumptions about the system properties (state transition rates, relative pathway efficiencies, *etc.*) and identifies several conditions to be met for wrpSL. 1) Reversibility of pathway steps. In fact, only the first pathway step must be reversible, whereas reversibility of the second pathway steps enables additional genetic scenarios. 2) Possibility of kinetic trapping of an intermediate state of the pathway when both the backward and forward reactions are compromised. The trapping *per se* can be detrimental due to blockage of cell signaling, sequestering an essential compound, or toxicity. We have assumed lethal toxicity in our model. 3) The possibility of rescue by a parallel compensatory pathway may not be strictly required, but highlights the applicability of this concept to non-essential pathways.

The mathematical model is validated by the experimentally observed recombination-dependent SL of the *srs2 rad54* double mutant in budding yeast [35] (Figure 3, Figure S1 in Text S1, Figure 6, row 5). Srs2-defective cells are unable to reverse Rad51-ssDNA filaments. These Rad51-ssDNA filaments represent toxic intermediates that accumulate in the cell due to kinetic trapping and interfere with cell viability. The key functions of the Rad54 protein are to assist in DNA strand invasion and allowing DNA synthesis off the invading 3′-end [36]. Hence, in the *srs2 rad54* double mutant Rad51-ssDNA filaments and/or D-loops may accumulate forming a toxic intermediate that leads to cell death (Figure S1 in Text S1; Figure 3 green pair and Figure 6, row 5). This interpretation is supported by the observation that lethality in this double mutant is suppressed by a defect in Rad51-ssDNA filament formation (mutations in *RAD51*, *RAD55*, *RAD57*, or *RAD52*) [49] (see Figure S1 in Text S1), what has been termed recombination-dependent lethality. Preventing Rad51-ssDNA filament formation allows bypass of recombination by alternative means of DNA repair (for DSBs: Nonhomologous endjoining or single-strand annealing; for gaps: Translesion synthesis or fork regression [23]; see Figure S1 in Text S1). The recombination-dependent lethality of *srs2 rad54* is not unique and is also found in additional double mutants in recombinational repair genes including the double mutants *mph1 mus81*, *mph1 mms4*, *srs2 sgs1 and sgs1* (or *top3* or *rmi1*) and *mus81* (or *mms4*) which likely reflect additional examples of wrpSL possibly involving different toxic intermediates [35], [46]–[53]. As discussed in detail in Section S1 in Text S1, the synthetic lethalities involving *sgs1* are more complex, because of the multiple roles of Sgs1-Top3-Rmi1 in HR, and could be caused also by other mechanisms of SL.

Further modeling revealed additional genetic conditions including overexpression of specific pathway enzymes that are predicted to lead to wrpSL (Figure 6). The mathematical modeling also reveals the importance of reversible pathway steps, which are validated by genetic and biochemical experiments in yeast [23]–[25]. First, the existence of reversible pathway steps does not affect normal pathway progression (Figure 6, rows 1–3). Second, reversible pathway steps allow much more efficient and timely use of compensatory pathways (Figure 6, row 6). Third, reversible pathways coupled with compensatory pathways avoid lethality of single mutations (Figure 6, row 7). The existence of reversible intermediates in HR, and possibly other molecular pathways, has been proposed to increase the robustness of the overall DNA repair system [23]–[25], and here we provide quantitative modeling evidence and formal analysis of this assertion.

**Figure 8:**
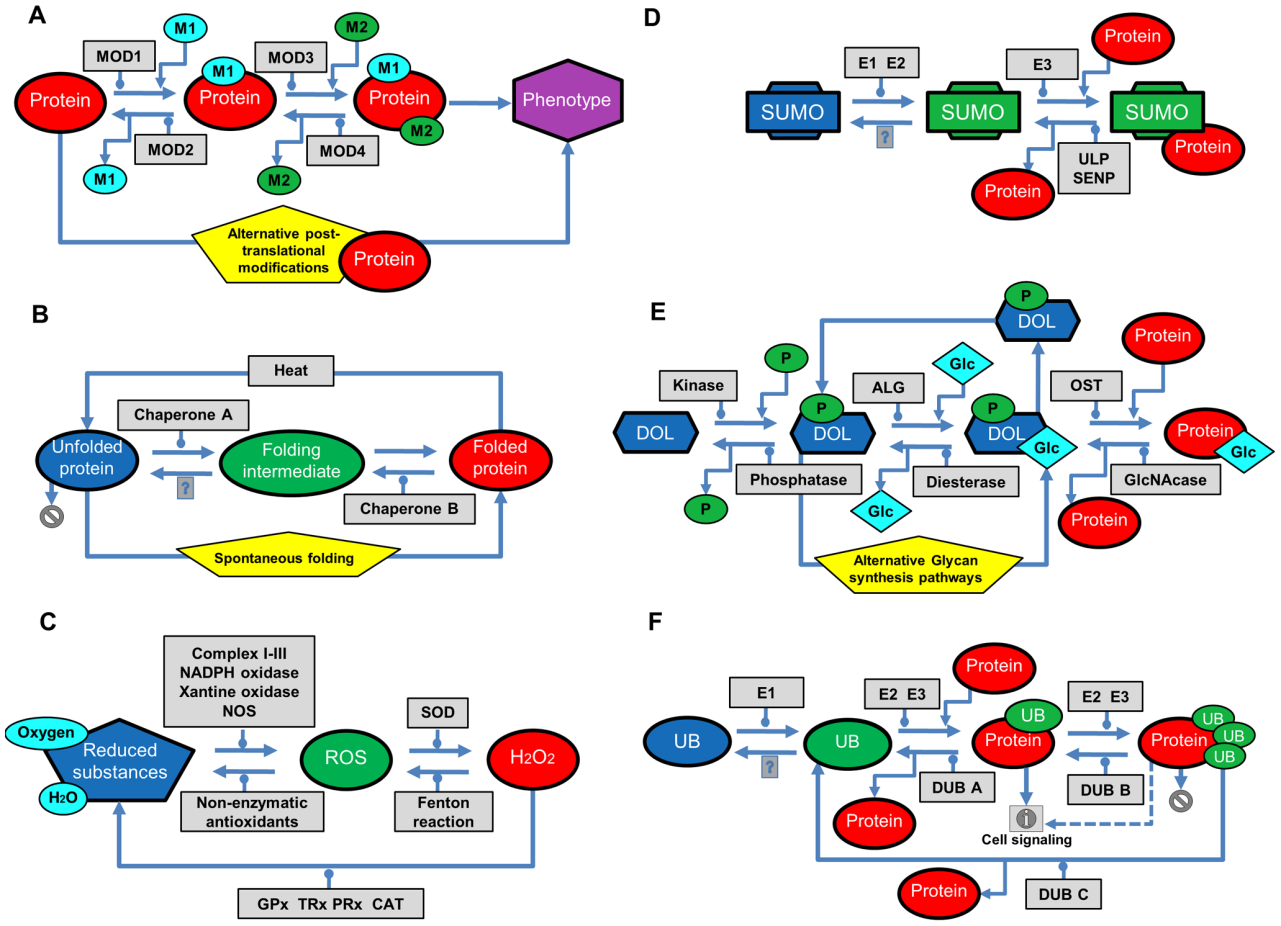
Examples of molecular processes with alternative pathways and potential to kinetic trap. A) Multiple post-translational protein modifications (phosphorylation followed by ubiquitination; acetylation followed by phosphorylation; sumotargeted ubiquitination, *etc.*). M1 and M2 represent two types of post-translational modifications. MOD1–4 represent enzymes catalyzing the reactions. Kinetic trapping of an intermediate modification can drastically disturb the balance between signaling pathways (*e.g.* #23 in Figure 2) B) Protein folding control by chaperones. Protein folding is controlled by chaperones (#18 in Figure 2) and may generate partially unfolded proteins as toxic intermediates which are subject to degradation [55]. Regulation of protein folding homeostasis is essential for protein pool control [56]; C) Lack of balance between production and detoxification of ROS leads to significant increases or drop in ROS levels that can be detrimental for cell signaling [57]–[59] (#19 in Figure 2). D) Coordinated sumoylation-desumoylation is important for proper signal propagation [60]–[62]. E) Glycan biosynthesis and protein glycosylation depend on the availability of common carrier dolichol phosphate (P-DOL). Correct recycling of P-DOL is important for sustaining the pool and utilization of this carrier in other glycans biosynthesis pathways. Kinetic trapping can consume the pool of P-DOL and perturb cell signaling [63], [64] (#2, 3 and 26 in Figure 2) F) Protein ubiquitylation is not only a tagging signal for degradation, but also involved in signaling. The correct tuning between two functions of ubiquitylation depends on the type and the length of ubiquitin (UB) polymer transferred to the protein and at the ubiquitylation site [65]. Monoubiquitylated proteins participate in signal transduction [66], [67], whereas K48-polyubiquitylated proteins are redirected to the proteasome for proteolysis. Thus, the balance between protein homeostasis and ubiquitin-dependent signaling is essential [68].

An important question is how general wrpSL might be or whether it is an idiosyncrasy of the recombinational repair pathway. Even if wrpSL were restricted to HR, this concept provides significant potential application in anti-cancer therapy. However, there is considerable evidence that many molecular pathways include reversible steps catalyzed by different enzymes in the forward and backward directions (see Figure 8). Any of those processes can be theoretically trapped into one of their intermediate states if two regulators of forward and backward steps are inactive. In these cases, the accumulating intermediate might be toxic, block proper signal propagation or prevent resource recycling. Focusing on three examples of reversible protein modifications (phosphorylation by Cdc5/dephosphorylation by Cdc14, sumoylation by Slx5–Slx8/desumoylation by Ulp1, Nup60, ubiquitylation by Rad6–Rad18/deubiquitylation by Bre5, Ubp3 or degradation dependent on Doa1, Rpn6; see Figure S6 in Text S1 for details), we found ample evidence in published genetic interaction data that are consistent with the wrpSL mechanism. These examples have not been fully explored, but are consistent with the wrpSL concept and amenable to test specific predictions.

In summary, genetic and biochemical data strongly support the significance of the wrpSL mechanism in HR, and existing data are consistent with the notion that wrpSL could be a general, widely applicable type of genetic interaction. This may refine our understanding of relationships between gene products and will help to improve pathway reconstruction. In particular, our mathematical modeling provides a conceptual framework for guiding systematic exploitation of mutations and changes in the expression profiles of HR genes and potentially genes of other pathways to induce SL.

## Materials and Methods

### Model formalism and model solution

The simplest mathematical model of Figure 3 was converted into a set of linear ordinary differential equations using the standard chemical kinetics formalism. The steady state model properties were analyzed analytically and exemplified with numerical simulations. Classification of the pathway states according to the extreme (large or small) values of the control parameters and the corresponding asymptotic solutions follow the methodology of the asymptotology of reaction networks [54].

### Numerical simulations

All numerical simulations were performed using SBTOOLBOX package for Matlab (Section S4 in Text S1).

## Supporting Information

Text S1 Text S1 contains Figure S1–S6, Section S1–S4, and Table S1.
(PDF)
