# Supplementary material for: Synthetic Lethality between Gene Defects Affecting a Single Non-essential Molecular Pathway with Reversible Steps
Source: PLoS Comput Biol. 2013 Apr 4;9(4):e1003016. doi: 10.1371/journal.pcbi.1003016 (PMC3617211; doi:10.1371/journal.pcbi.1003016)
Supplement: Text S1 — contains Figure S1–S6, Section S1–S4, and Table S1. (PDF) [file pcbi.1003016.s001.pdf]

Text S1

Synthetic lethality between gene defects affecting a single non-essential molecular pathway with reversible steps

Andrei Zinovyev[1,2,3*], Inna Kuperstein[1,2,3], Emmanuel Barillot[1,2,3] , and Wolf-Dietrich Heyer[4#]

[1] Institut Curie, 26 rue d'Ulm, F-75248 Paris, France

[2] INSERM, U900, Paris, F-75248, France

[3] Mines ParisTech, Fontainebleau, F-77300, France

[4] Departments of Microbiology & Molecular Genetics and of Molecular & Cellular Biology, University of California, Davis, One Shields Avenue, Davis, CA 95616-8665, USA

*These two authors contributed equally to the work

# Corresponding author:

Tel. (530) 752-3001 FAX (530) 752-3011 Email wdheyer@ucdavis.edu

TABLE OF CONTENTS

Figure S1	Page 3
Figure S2	Page 5
Figure S3	Page 6
Figure S4	Page 8
Figure S5	Page 10
Figure S6	Page 12
Section S1	Page 16
Section S2	Page 21
Section S3	Page 27
Section S4	Page 29
Table S1	Page 31

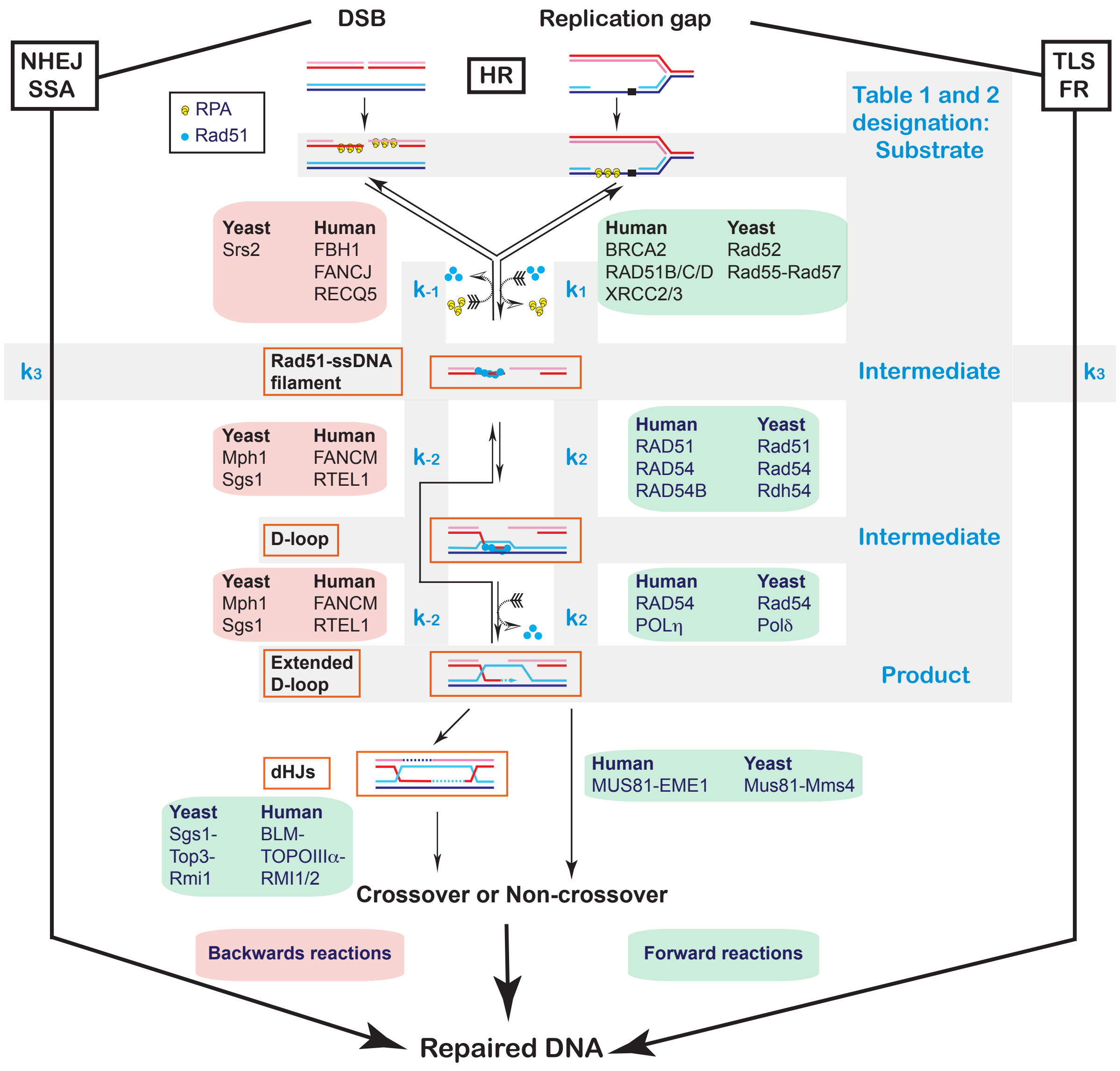

Figure S1. Homologous Recombination (HR) DNA repair pathway. The substrate for homologous recombination (HR) is RPA-covered ssDNA, as it occurs at processed double-strand breaks (DSB) (left) or in replication-associated gaps (right). The ensuing pathways steps are elaborated for DSB repair, but analogous steps exist for gap repair. The proteins from the yeast *S. cerevisiae* and humans catalyzing individual steps in the forward (green shaded) and backwards (red shaded) reactions are indicated. The designation of the substrate, intermediates, and products, as well as reaction steps (k_1, k_{-1}, k_2, k_{-2}, k_3) relating to the models in Figures 3, 4, 6, and

Figure S2 in Text S1 are indicated in light blue with grey shading. It is presently unclear, which of the intermediates in the *srs2 rad54* double mutant, Rad51-ssDNA filament or D-loops or both, are toxic but this has no effect on the present discussion. The reversion of extended D-loops channels recombination events to synthesis-dependent strand annealing (not depicted in Figure S1 in Text S1). In this case, the displaced invading strand anneals with the second end of the DSB to always generate non-crossover outcomes. DSBs can also be repaired by non-homologous endjoining (NHEJ) or single-strand annealing (SSA), whereas replication gaps could be processed by translesion synthesis (TLS) or fork regression (FR). These compensatory pathways are labeled as k_3 in the modeling diagrams.

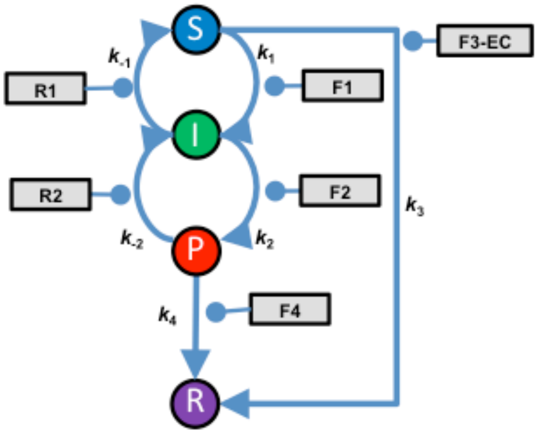

Figure S2. Abstract representation of the recombinational DNA repair pathway with a final irreversible step. A modification of the simplest toy model (Figure 3) introduing a last irreversible step from Figure S1 in Text S1 that enables kinetic proof-reading. In this case the repair product **P** is irreversibly transformed to the final repaired state **R**. The scheme represents a simplified version of the pathway depicted in Figure S1 in Text S1 using the same abbreviations. Dynamic states **S**, **I**, **P** represent DNA damage substrate (**S)**, toxic intermediate (**I**), and the product of repair (**P**). **F1** (*e.g.* Rad51), **F2** (*e.g.* Rad54), **R1** (*e.g.* Srs2), **R2** (*e.g.* Mph1) are enzymes in the main pathway, and **F3-EC** represent enzymes in the compensatory pathway.

Model Figure 3

without irreversible last step

Model Figure S2 in Text S1

with irreversible last step

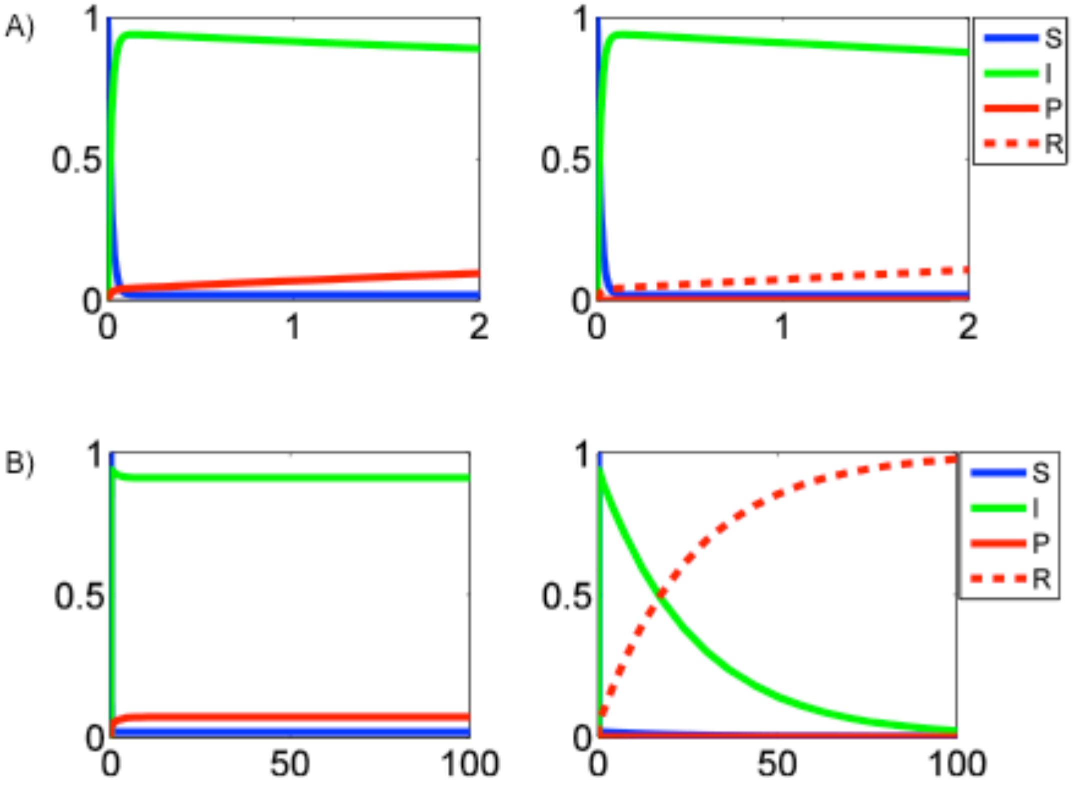

Figure S3. Illustrating the difference between the simplest toy model (Figure 3) and the model with a last irreversible pathway step (Figure S2 in Text S1). The simulation was performed for the F2🡇 F1🡅 scenario (Figure 6, line 13). Simulations for the toy model are shown on the left, simulations for the model with a last irreversible step are shown on the right. Top: The dynamics of the two models are practically indistinguishable in the scale of 2 time units. Bottom: The dynamics of the two models is very different in the scale of 100 time units. The plots are insensitive to several order of magnitude changes in the value of the parameter k_4. Dynamic states **S**, **I**, **P, R** represent DNA damage substrate (**S**), toxic intermediate (**I**), and the product of repair (**P**) and the final repaired state (**R**). **Role of the last irreversible step in the**

model: The difference between two models of homologous recombination DNA repair presented in Figure 3 and S2 is the presence of an irreversible step to a final repaired product in the latter. In the case of DNA repair, this might be justified: the enzymes regulating the backward reactions should not be able to cause DNA damage. However, in other contexts, the relevance of a last irreversible step might be less obvious (see Discussion and Figure 8). The presence of a last irreversible step modifies several but not all cell fates in Figure 6 (verified by comparing numerical simulations for the two models and varying the parameter k_4). First, the normal wild-type models are not affected. Second, the bpSL and wrpSL scenario are not affected. Those genetic scenarios whose cell fates can be reversed in the presence of the last irreversible step are shown as hexagons in Figure S4A in Text S1 and indicated in Figure S4B in Text S1. In these cases, the effect of timing starts playing the most crucial role and the simple analysis of only steady state values is not sufficient. At a short time scale, the dynamics of the two models can be practically indistinguishable, while being very different at longer time scales (see above). In particular, this is the case for scenario 8 (F2↓) and scenario 13 of Figure 6 (F2↓F1↑) (see also Figure S4 in Text S1). Thus, for making predictions from these simulations, one needs to estimate more precisely the T_{dam} and T_{tox} delays (time intervals during which the DNA damage and the toxicity can be tolerable) that become crucial parameters in the cell fate decisions.

A

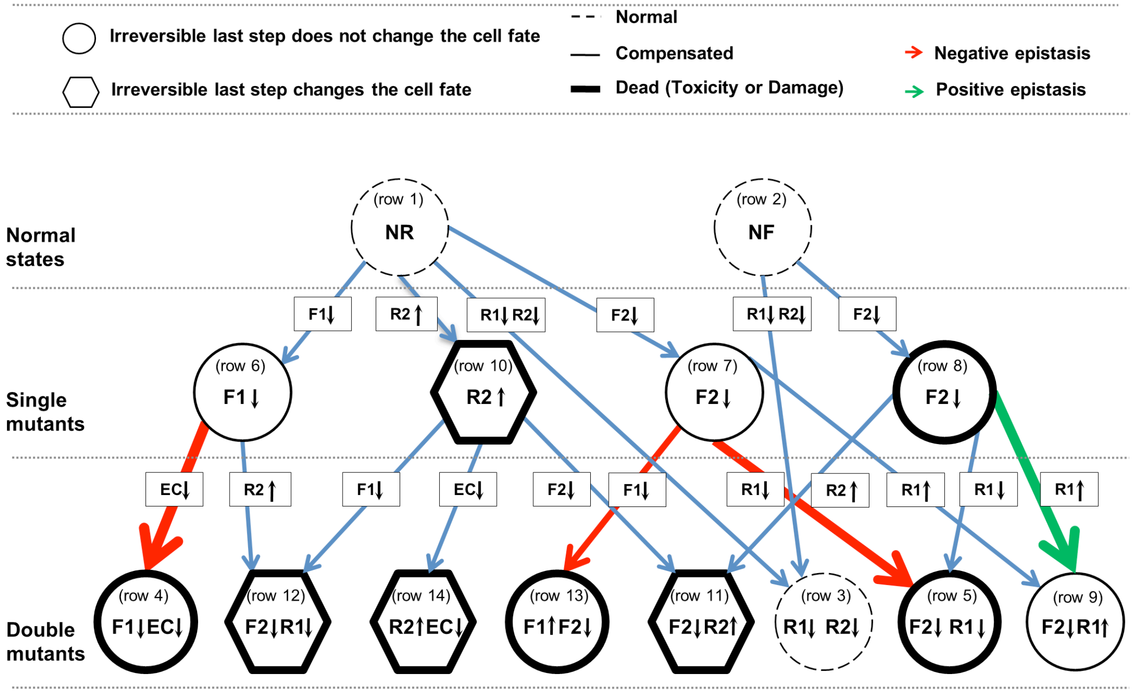

B

Simulation (Table 3)	Type	Fitness	Mutant	Last irreversible step changes cell fate
(1) NR	N	0.98	-	no
(2) NF	N	0.91	-	no
(3) R1↓R2↓	N	1.00	double	no
(4) F1↓EC↓	DD	0.00	double	no
(5) F2↓R1↓	DT	0.02	double	no
(6) F1↓	C	0.98	single	no
(7) F2↓	C	0.60	single	no
(8) F2↓	DT	0.00	single	no
(9) F2↓R1↑	C	0.76	double	no
(10) R2↑	DT	0.09	single	yes
(11) F2↓R2↑	DT	0.00	double	yes
(12) F1↓R2↓	DD	0.07	double	yes
(13) F1↑F2↓	DT	0.27	double	no
(14) R2↑EC↓	DT	0.08	double	yes

Figure S4. Relationships between wild type, single and double mutant model modifications. The probability of complete repair of DNA damage can serve as a measure of **fitness** for the different genetic models. For genetic scenarios affecting two genes the strength of the interaction between knock-outs or overexpression conditions are measured by the deviation from the multiplicative model $\varepsilon = W_{ab} - W_a W_b$, where W_a and W_b are the fitness values for single condition

(knockout or overexpression) and W_{ab} is the fitness with a knockout and overexpression. **A)** The nodes correspond to the simulations from Figure 6. The node border style shows viability as defined from the steady state of repaired DNA (value P^s, formula (4)). Hexagonal nodes denote those lethal scenarios which can be rescued by the addition of the last irreversible pathway step (see Figure S2 in Text S1). Edges of red color show strong negative and of green color strong positive genetic interactions; B) A summary of numerical and label attributes for the simulations shown in A). They show that in the examples from Figure 6 there are three cases of strong negative interactions (between-pathway synthetic lethality F1↓ EC↓, within-reversible-pathway synthetic lethality F2↓R1↓ and within-pathway synthetic dosage lethality F2↓F1↑) and one example of strong positive interaction (synthetic dosage interaction F2↓ R1↑).

	Pathway state		Enzyme change	Model diagram	Dynamic plot	Description and cell fates
	Normal	Mutant				
**Between Pathways Synthetic Lethality (bpSL)**						
**1**	Normal Robust (NR) or Normal Fragile (NF)	Death from DNA Damage (DD)	**R1↑ and EC↓**	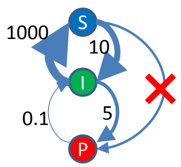	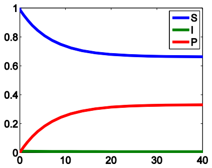	This is a case of synthetic dosage synthetic lethality (SDL) leading to the inefficiency of the first pathway step, as in Figure 6, row 4.
**2**	Normal Robust (NR) or Normal Fragile (NF)	Death from Toxicity (DT)	**F2↓ and EC↓**	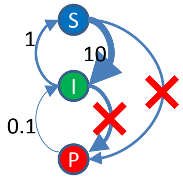	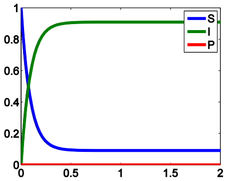	This is an alternative example of parallel pathway synthetic lethality (compare to Figure 7, row 14) which mechanism of action is analogous.
**Within Reversible Pathway Synthetic Lethality (wrpSL)**						
**3**	Normal Robust (NR) or Normal Fragile (NF)	Death from Toxicity (DT)	**R2↑ and R1↓**	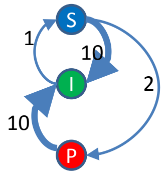	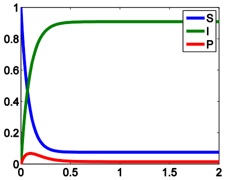	This is a case of synthetic dosage synthetic lethality (SDL) equivalent by its effect to the scenario in Figure 6, row 5.

			**Dosage effects**			
**4**	Normal Robust (NR) or Normal Fragile (NF)	Death from DNA damage (DD) or Toxicity (DT)	**R2↑ and R1↑**	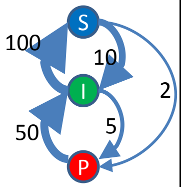	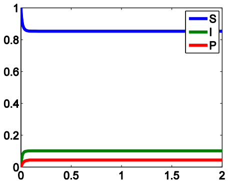	Double overexpression of backward enzymes leads to the cell death (DD or DT depending on the ratio of the R1 and R2 steps).
**5**	Normal Robust (NR) or Normal Fragile (NF)	Death from Toxicity (DT)	**R2↑ and F1↑**	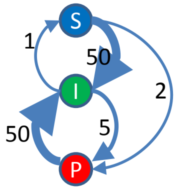	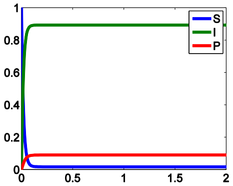	Double overexpression of R2 and F1 leads to the cell death by the mechanism equivalent to the scenario shown in Figure 7, row 13.

Figure S5. Additional modeling scenarios extending Figure 6. Pathway states depicted as **Normal states** as Normal Robust (***NR***) and Normal Fragile (***NF***); **Mutant states** as death from DNA damage (***DD***) and death from toxicity (***DT***). **Dynamic plots** show prediction of model for evolution of substrate (**S**), intermediate (**I**) and product (**P**) amounts over time corresponding to the choice of kinetic parameters shown on the **Model diagram**. (**X-axis**)-time, (**Y-axis**)-substances level. **F1**, **F2**, **R1**, **R2**, and **EC** refer to the enzymes catalyzing the two forward and two backwards reactions as well as the compensatory pathway, respectively (see Figure 2). (↓)-complete knock-down or mutational loss of function; (↑)-over-expression.

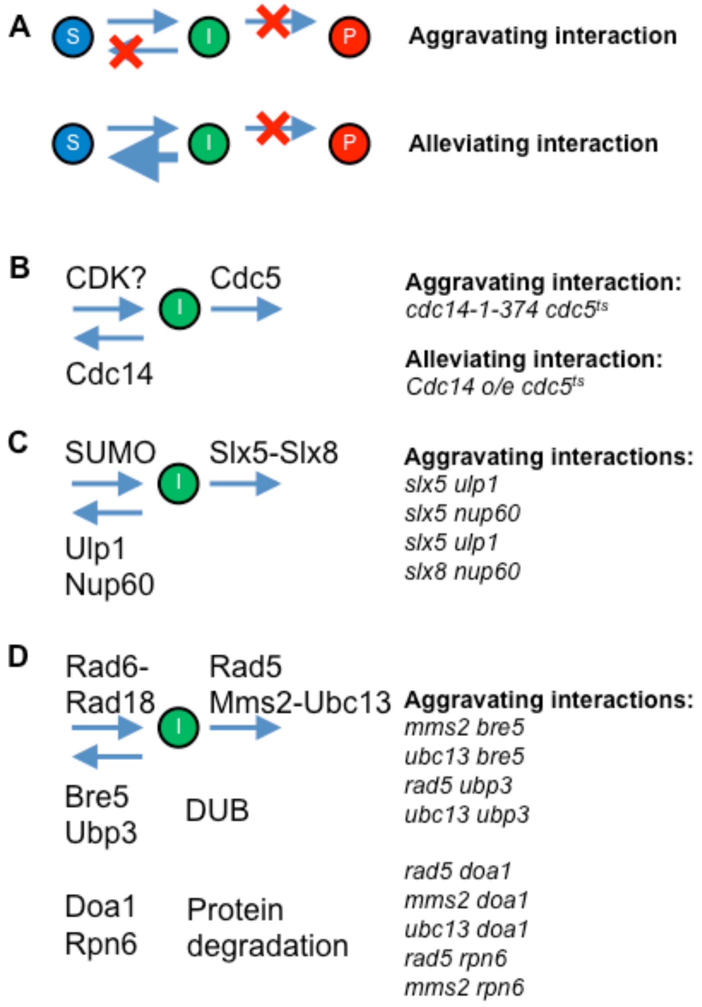

Figure S6. Potential examples of within-reversible-pathway Synthetic Lethality (wrpSL). A) Reduced scheme of wrpSL indicating a first reversible reaction and a second dependent reaction making specific predictions about synthetic negative or aggravating interactions of mutations (loss of function or hypomorphs) and synthetic positive or alleviating interactions when the enzyme of the backwards reaction is overexpressed. The reversibility of step 2 is not required for wrpSL. **S**: substrate, **I**: intermediate, **P**: product. A red cross indicates a mutation and an arrow with increased weight means overexpression. B) Genetic interaction between *CDC5* (Polo kinase) and *CDC14* (FEAR complex phosphatase) [1,2]. Cdc5 Polo kinase is dependent on prior phosphorylation (priming) by other kinases, often cyclin-dependent kinases (CDK), using its Polo

domain, a phospho-epitope binding motif, to bind to the primed protein substrate [3]. The phosphatase Cdc14 also functions in cell cycle regulation in conjunction with Cdc5 [4]. Mutations in *CDCD14* and *CDC5* were shown to have a synthetic negative interaction, an observation not [1] easily explained by a mechanism proposing that Cdc5 and Cdc14 target the same residue for phosphorylation or dephosphorylation. Such a mechanism would predict mutual suppression. The wrpSL mechanism, however, is consistent with the observed negative interaction. Importantly, this system also fulfills a further prediction of wrpSL, in that Cdc14 overexpression shows an alleviating interaction with a *cdc5* mutation [2]. This example highlights that wrpSL can also be applied to essential pathways with hypomorphic mutations (Figure 5). The wrpSL mechanism involving a toxic intermediate is also consistent with the model and interpretation of the finding that overexpression of certain substrates (Pho4, Gsy1, Gsy2, Gcn4) shows an aggravating effect in a deletion of their kinase *PHO85* [5,6]. C) Genetic interactions between the SUMO-targeted Ubiquitin ligase (StUbL) encoded by *SLX5-SLX8* with *ULP1* (desumoylation protease) or *NUP60* (regulator of desumoylation) [7-12]. StUbLs are enzymes that ubiquitylate their substrates being targeted by prior sumoylation [13]. In yeast, Slx5-Slx8 constitute a heterodimeric StUbL [14], and both genes show a negative interaction with the SUMO-protease Ulp1 and the desumoylation regulator Nup60, a protein associated with the nuclear pore complex [7-12] (see Figure 8A, D). This genetic interaction could identify the specific SUMO-ligase working with a particular StUbL. D) Genetic interactions of *RAD5, MMS2, UBC13* encoding a poly-ubiquitin ligase complex with *BRE5* and *UBP3* (deubiquitinases, DUB) [7,15], as well as with *DOA1* and *RPN6*, which encode proteins involved in protein degradation [10,16]. Rad6-Rad18 monoubiquitin ligase primes PCNA for polyubiquitination by Rad5-Mms2-Ubc13 [17]. Potentially other substrates are involved as well or different priming monoubiquitin

ligases. It is interesting to note that *RAD5, MMS2,* and *UBC13* show negative genetic interactions with two different ubiquitin proteases (DUB), Bre5 and Ubp3 [10,16], possibly indicating the involvement of different toxic intermediates. Instead of reversing the toxic intermediate by de-ubiquitylation, it is also possible that the intermediate is degraded, and we note that *RAD5, MMS2,* and *UBC13* show a consistent negative interaction with *DOA1* and *RPN6*, which are involved in protein degradation [7,15] (see Figure 8F). The different negative interactions of the Rad5-Mms2-Ubc13 polyubiquitin ligase may suggest that different toxic intermediates accumulate depending on the particular DUB or protein degradation adaptor and may help identify them.

Supplemental References

1. Rahal R, Amon A (2008) The Polo-like kinase Cdc5 interacts with FEAR network components and Cdc14. Cell Cycle 7: 3262-3272.
2. Jaspersen SL, Charles JF, Tinker-Kulberg RL, Morgan DO (1998) A late mitotic regulatory network controlling cyclin destruction in *Saccharomyces cerevisiae*. Molecular Biology of the Cell 9: 2803-2817.
3. Archambault V, Glover DM (2009) Polo-like kinases: conservation and divergence in their functions and regulation. Nature Reviews Molecular Cell Biology 10: 265-275.
4. Segal M (2011) Mitotic exit control: A spae and time odyssey. Current Biology 21: R857-R859.
5. Sopko R, Huang D, Preston N, Chua G, Papp B, et al. (2006) Mapping pathways and phenotypes by systematic gene overexpression. Molecular Cell 21: 319-330.
6. Boone C, Bussey H, Andrews BJ (2007) Exploring genetic interactions and networks with yeast. Nature Reviews Genetics 8: 437-449.
7. Costanzo M, Baryshnikova A, Bellay J, Kim Y, Spear ED, et al. (2010) The genetic landscape of a cell. Science 327: 425-431.
8. Li T, Mullen JR, Slagle CE, Brill SJ (2007) Stimulation of in vitro sumoylation by Slx5-Slx8: Evidence for a functional interaction with the SUMO pathway. DNA Repair 6: 1679-1691.
9. Burgess RC, Rahman S, Lisby M, Rothstein R, Zhao XL (2007) The slx5-slx8 complex affects sumoylation of DNA repair proteins and negatively regulates recombination. Molecular and Cellular Biology 27: 6153-6162.

10. Collins SR, Miller KM, Maas NL, Roguev A, Fillingham J, et al. (2007) Functional dissection of protein complexes involved in yeast chromosome biology using a genetic interaction map. Nature 446: 806-810.
11. Wilmes GM, Bergkessel M, Bandyopadhyay S, Shales M, Braberg H, et al. (2008) A Genetic Interaction Map of RNA-Processing Factors Reveals Links between Sem1/Dss1-Containing Complexes and mRNA Export and Splicing. Molecular Cell 32: 735-746.
12. Pan XW, Ye P, Yuan DS, Wang XL, Bader JS, et al. (2006) A DNA integrity network in the yeast Saccharomyces cerevisiae. Cell 124: 1069-1081.
13. Perry JJP, Tainer JA, Boddy MN (2008) A SIM-ultaneous role for SUMO and ubiquitin. Trends in Biochemical Sciences 33: 201-208.
14. Prudden J, Pebernard S, Raffa G, Slavin DA, Perry JJP, et al. (2007) SUMO-targeted ubiquitin ligases in genome stability. Embo Journal 26: 4089-4101.
15. Dixon SJ, Fedyshyn Y, Koh JLY, Prasad TSK, Chahwan C, et al. (2008) Significant conservation of synthetic lethal genetic interaction networks between distantly related eukaryotes. Proceedings National Academy of Sciences USA105: 16653-16658.
16. Lis ET, Romesberg FE (2006) Role of *DOA1* in the *Saccharomyces cerevisiae* DNA damage response. Molecular and Cellular Biology 26: 4122-4133.
17. Unk I, Hajdu I, Blastyak A, Haracska L (2010) Role of yeast Rad5 and its human orthologs, HLTF and SHPRH in DNA damage tolerance. DNA Repair 9: 257-267.

Section S1

Mechanism of recombination and single pathway synthetic lethal interactions.

Homologous recombination (HR) is an important mechanism to maintain genome integrity [1]. HR plays a fundamental role in meiosis generating genetic diversity and ensuring accurate chromosome segregation. HR is a central DNA repair pathway of complex DNA damage such as double-stranded breaks (DSB), interstrand crosslinks (ICL), and single-stranded DNA gaps. The stalling or collapse of replication forks requires HR to complete DNA synthesis during S-phase in cycling cells. HR is of particular importance in cancer biology for its dual significance in tumor suppression and cancer therapy [2]. Key HR factors, such as BRCA1, BRCA2, RAD51C, and BLM, have been identified as tumor suppressors (Table S1). Moreover, induction of DNA damage in cancer therapy by ionizing radiation (IR), interstrand crosslinks, topoisomerase inhibitors and alkylating agents leads to DNA damage that are HR substrates.

The key reaction of HR is homology search and DNA strand invasion catalyzed by the Rad51-ssDNA filament (Figure S1 in Text S1) [1]. In order to assemble Rad51-ssDNA filaments, the DNA damage (DSB or gaps) must be processed to reveal ssDNA of sufficient length. The eukaryotic ssDNA binding protein RPA immediately covers available ssDNA, and mediator proteins such as the Rad51 paralogs Rad55-Rad57 and Rad52 in yeast (Rad51B/C/D, XRCC2/3, BRCA2 in humans) are required for Rad51-ssDNA filament formation. After strand invasion, Rad54 is required to turnover the Rad51-dsDNA product complex to allow access by DNA polymerases to the 3'-end of the invading strand. The ensuing DNA synthesis restores the missing DNA leading to the formation of junction intermediates that require either one of two pathways for processing to repaired end products. One pathway, double Holliday junction

dissolution, involves the helicase Sgs1 (human BLM) in association with the type 1A topoisomerase Top3 (human TOPOIIIα) and the specificity factor Rmi1 (human RMI1/2). The second pathway involves cleavage of junction molecules and is defined by the endonuclease Mus81-Mms4 (human MUS81-EME1) [3]. Hence, the recombination pathway bifurcates in this last step into two pathways, one Sgs1/BLM-dependent, the other Mus81-dependent (Figure S1 in Text S1).

While the above discussion summarizes accurately the forward reactions in the HR pathway, it has become clear that dedicated motor proteins and DNA helicases catalyze specific backwards reactions that either antagonize recombinational repair outright or channel intermediates into specific sub-pathways (Figure S1 in Text S1). The paradigm for an anti-recombinase is the *Saccharomyces cerevisiae* Srs2 helicase, which serves to repress recombination [4-6]. Biochemical analysis uncovered a satisfying mechanism for anti-recombination by showing that Srs2 dissociates Rad51 from ssDNA [7,8]. Candidates for equivalent activities in humans are FBH1, FANCJ, and RECQ5 [9-11]. This identifies the Rad51-ssDNA filament as a reversible intermediate in recombination (Figure S1 in Text S1, corresponds to I in Figure 2). Another reversible intermediate is the D-loop (Figure S1 in Text S1, corresponds to I in Figure 2), which is formed by the Rad51-ssDNA filament and dissociated by the Mph1 protein [12] and possibly Sgs1 [13-15]. In humans, FANCM and RTEL1 exhibit such an activity [16,17]. D-loop reversal is anti-recombinogenic *per se*. However, reversion of an extended D-loop (Figure S1 in Text S1 after DNA synthesis from the invading strand) does not prevent recombination, but channels recombination to the synthesis-dependent strand annealing (SDSA) pathway, which always lead to a non-crossover outcome. In fact, mutations in RTEL1 show the expected increase crossover

formation during *Caenorhabditis elegans* meiosis [18]. Thus the extended D-loop could be viewed as a reversible intermediate, but the reaction could equally be a direct forward step to SDSA (Figure S1 in Text S1, corresponds to P in Figure 2).

Synthetic lethality between various single gene mutations within the recombinational DNA repair pathway has been observed in budding yeast. Of particular interest are cases where the synthetic lethality was demonstrated to be recombination-dependent, meaning the synthetic lethality was suppressed by a third mutation disabling an early stage of the recombination pathway. Examples include the following double mutants: *srs2 rad54, mph1 mus81* or *mph1 mms4, srs2 sgs1* [19-24], and the synthetic lethal interaction between *mus81* or *mms4* and *sgs1* or *top3* or *rmi1* in yeast [25] with similar observations being reported in *Drosophila* [26]. The recombination-dependent synthetic lethality involving Sgs1 are more complex, because of the multiple roles of Sgs1-Top3-Rmi1 in DSB repair including DSB resection, joint molecule disruption, and double Holliday junction dissolution (Figure S1 in Text S1) [27]. Both Mus81-Mms4 and the Sgs1-Top3-Rmi1 complex function in the processing of late recombination intermediates (Figure S1 in Text S1), the genetic situation resembles within-pathway synthetic lethality involving parallel forward pathways (Figure 2B2). In this case, the recombination pathway bifurcates into alternate routes of product formation (Figure S1 in Text S1). The twist is that a complete recombination defect in yeast is not lethal *per se*, suggesting that toxic intermediates, accumulating in the double mutant, cause lethality. Alternatively, the role of Sgs1 in disrupting joint molecules [13-15] could be the cause for the synthetic lethality with *mus81* [25], which would conform to the mechanism of within-reversible-pathway synthetic lethality. Likewise, it is unclear which role of Sgs1 leads to its synthetic lethality with Srs2, D-loop disruption or double Holliday junction dissolution. The

role of Sgs1 in DSB resection is quite redundant with other helicases and nucleases [28,29], and this function does not appear to limit recombinational repair, as suppression of late defects in for example Rad54, Mus81-Mms4 would have been expected by mutations in Sgs1.

Supplemental References

1. Li X, Heyer WD (2008) Homologous recombination in DNA repair and DNA damage tolerance. Cell Research 18: 99-113.
2. Helleday T (2010) Homologous recombination in cancer development, treatment and development of drug resistance. Carcinogenesis 31: 955-960.
3. Schwartz EK, Heyer WD (2011) Processing of joint molecule intermediates by structure-selective endonucleases during homologous recombination in eukaryotes. Chromosoma 120: 109-127.
4. Aboussekhra A, Chanet R, Zgaga Z, Cassier Chauvat C, Heude M, et al. (1989) *RADH*, a gene of *Saccharomyces cerevisiae* encoding a putative DNA helicase involved in DNA repair. Characteristics of *radH* mutants and sequence of the gene. Nucleic Acids Research 17: 7211-7219.
5. Schiestl RH, Prakash S, Prakash L (1990) The *SRS2* suppressor of *rad6* mutations of *Saccharomyces cerevisiae* acts by channeling DNA lesions into the *RAD52* DNA repair pathway. Genetics 124: 817-831.
6. Aguilera A, Klein HL (1988) Genetic control of intrachromosomal recombination in Saccharomyces cerevisiae. I. Isolation and genetic characterization of hyper-recombination mutations. Genetics 119: 779-790.
7. Veaute X, Jeusset J, Soustelle C, Kowalczykowski SC, Le Cam E, et al. (2003) The Srs2 helicase prevents recombination by disrupting Rad51 nucleoprotein filaments. Nature 423: 309-312.
8. Krejci L, Van Komen S, Li Y, Villemain J, Reddy MS, et al. (2003) DNA helicase Srs2 disrupts the Rad51 presynaptic filament. Nature 423: 305-309.
9. Fugger K, Mistrik M, Danielsen JR, Dinant C, Falck J, et al. (2009) Human Fbh1 helicase contributes to genome maintenance via pro- and anti-recombinase activities. Journal of Cell Biology 186: 655-663.
10. Sommers JA, Rawtani N, Gupta R, Bugreev DV, Mazin AV, et al. (2009) FANCJ uses its motor ATPase to destabilize protein-DNA complexes, unwind triplexes, and inhibit RAD51 strand exchange. Journal of Biological Chemistry 284: 7502-7514.
11. Schwendener S, Raynard S, Paliwal S, Cheng A, Kanagaraj R, et al. (2010) Physical Interaction of RECQ5 Helicase with RAD51 Facilitates Its Anti-recombinase Activity. Journal of Biological Chemistry 285: 15739-15745.
12. Prakash R, Satory D, Dray E, Papusha A, Scheller J, et al. (2009) Yeast Mph1 helicase dissociates Rad51-made D-loops: implications for crossover control in mitotic recombination. Genes and Development 23: 67-79.

13. Oh SD, Lao JP, Hwang PYH, Taylor AF, Smith GR, et al. (2007) BLM ortholog, Sgs1, prevents aberrant crossing-over by suppressing formation of multichromatid joint molecules. Cell 130: 259-272.
14. Oh SD, Lao JP, Taylor AF, Smith GR, Hunter N (2008) RecQ helicase, Sgs1, and XPF family endonuclease, Mus81-Mms4, resolve aberrant joint molecules during meiotic recombination. Molecular Cell 31: 324-336.
15. Jessop L, Lichten M (2008) Mus81/Mms4 endonuclease and sgs1 helicase collaborate to ensure proper recombination intermediate metabolism during meiosis. Molecular Cell 31: 313-323.
16. Gari K, Decaillet C, Stasiak AZ, Stasiak A, Constantinou A (2008) The Fanconi anemia protein FANCM can promote branch migration of Holliday junctions and replication forks. Molecular Cell 29: 141-148.
17. Barber LJ, Youds JL, Ward JD, McIlwraith MJ, O'Neil NJ, et al. (2008) RTEL1 maintains genomic stability by suppressing homologous recombination. Cell 135: 261-271.
18. Youds JL, Mets DG, McIlwraith MJ, Martin JS, Ward JD, et al. (2010) RTEL-1 Enforces Meiotic Crossover Interference and Homeostasis. Science 327: 1254-1258.
19. Palladino F, Klein HL (1992) Analysis of mitotic and meiotic defects in *Saccharomyces cerevisiae* SRS2 DNA helicase mutants. Genetics 132: 23-37.
20. Heyer WD, Ehmsen KT, Liu J (2010) Regulation of homologous recombination in eukaryotes. Annual Reviews Genetics 44: 113-139.
21. Heyer WD, Li X, Rolfsmeier M, Zhang XP (2006) Rad54: the Swiss Army knife of homologous recombination? Nucleic Acids Research 34: 4115-4125.
22. St Onge RP, Mani R, Oh J, Proctor M, Fung E, et al. (2007) Systematic pathway analysis using high-resolution fitness profiling of combinatorial gene deletions. Nature Genetics 39: 199-206.
23. Gangloff S, Soustelle C, Fabre F (2000) Homologous recombination is responsible for cell death in the absence of the Sgs1 and Srs2 helicases. Nature Genetics 25: 192-194.
24. Maftahi M, Hope JC, Delgado-Cruzata L, Han CS, Freyer GA (2002) The severe slow growth of Delta srs2 Delta rqh1 in *Schizosaccharomyces pombe* is suppressed by loss of recombination and checkpoint genes. Nucleic Acids Research 30: 4781-4792.
25. Fabre F, Chan A, Heyer WD, Gangloff S (2002) Alternate pathways involving Sgs1/Top3, Mus81/Mms4, and Srs2 prevent formation of toxic recombination intermediates from single-stranded gaps created by DNA replication. Proceedings National Academy Sciences USA 99: 16887-16892.
26. Trowbridge K, McKim K, Brill SJ, Sekelsky J (2007) Synthetic lethality of *Drosophila* in the absence of the MUS81 endonuclease and the DmBlm helicase is associated with elevated apoptosis. Genetics 176: 1993-2001.
27. Klein HL, Symington LS (2012) Sgs1-The Maestro of Recombination. Cell 149: 257-259.
28. Mimitou EP, Symington LS (2008) Sae2, Exo1 and Sgs1 collaborate in DNA double-strand break processing. Nature 455: 770-774.
29. Zhu Z, Chung WH, Shim EY, Lee SE, Ira G (2008) Sgs1 helicase and two nucleases Dna2 and Exo1 resect DNA double-strand break ends. Cell 134: 981-994.

Section S2

A. Description and analysis of the simplest mathematical model of a pathway with reversible steps and a toxic intermediate.

In the mathematical description of the simplest DNA repair model, there are five kinetic rates (two kinetic constants k_1 and k_{-1} of forward and backward reactions for the step S↔I, two kinetic constants k_2 and k_{-2} of forward and backward reactions for the step I↔P and the kinetic constant k_3 of the compensatory step S→P) which completely define the dynamic properties of the model, *i.e.* its steady state values and the history of relaxation of the model variable values from the initial condition towards the equilibrium values. For simplicity, we assume all state transition rates in the model (Figure 3) to be linearly dependent on the dynamic variables [S], [I] and [P], where square brackets denote the probability of the corresponding molecular state (a number between 0 and 1). The following system of equations describes the time evolution of the model system:

$$\begin{cases} [\dot{S}] = -k_1 \cdot [S] + k_{-1} \cdot [I] - k_3 \cdot [S] \\ [\dot{I}] = k_1 \cdot [S] - k_{-1} \cdot [I] - k_2 \cdot [I] + k_{-2} \cdot [P] \\ [\dot{P}] = k_2 \cdot [I] - k_{-2} \cdot [P] + k_3 \cdot [S] \end{cases} \qquad (1)$$

where [S], [I] and [P] are assumed to be functions of time. Initially the DNA is assumed to be in its damaged state:

$$S(0) = 1.0,\ I(0) = 0.0,\ P(0) = 0.0 \qquad (2)$$

During time evolution, the probability to find the damaged DNA locus in one of the three states is $S(t)$, $I(t)$, $P(t)$, and $S(t)+I(t)+P(t) = 1.0$ at any moment of time.

Let us formulate the conditions on cellular viability. We assume that the cell can tolerate unrepaired DNA damage above a certain threshold $[S]_{leth}\in[0;1]$ only for a limited amount of time T_{dam} otherwise it loses viability. Let us also assume that the intermediate I is toxic for the cell and when its probability $[I]$ remains higher than some threshold value $[I]_{tox}\in[0;1]$ for a sufficiently long time period T_{tox} then the cell dies because of toxic stress.

Analysis of the steady state solution and cell viability conditions. The system (1) is sufficiently simple that it allows explicit analytical solution in a closed form. The steady state solution of (1) is

$$S^s = \frac{k_{-1}k_{-2}}{A}, I^s = \frac{k_{-2}(k_1 + k_3)}{A}, P^s = \frac{k_2 k_1 + k_3(k_2 + k_{-1})}{A}, \qquad (3)$$

where $A = k_{-2}(k_1 + k_3) + k_3(k_2 + k_{-1}) + k_1 k_2 + k_{-1}k_{-2}$.

In our interpretation of synthetic interactions, the incomplete knock-down of one of the enzymes F1, F2, R1, R2, or EC (Figure 3) is modeled by setting the corresponding kinetic rate constant k_i small but not to zero: $k_i \neq 0$, while the complete knock-out is modeled by setting the corresponding rate constant to zero $k_i = 0$. Technically, the difference between even infinitely small values of kinetic rates and zero values in the model analysis is the following. Introducing a zero parameter can qualitatively change the type of the steady state solution. Hence, some conclusions about the effect of knocking down enzymes can be derived from the analytical treatment of the steady state. If instead of zero values, arbitrarily small kinetic rates are used, then it might create very long relaxation times towards the steady state. If the relaxation time is very long then it is equivalent to the existence of a "metastable" state of the system from the point of

view of practical interpretation of the results. Hence, using very small kinetic rates requires studying the dynamics of the system and comparing the relaxation times with the time of the experiment or duration of the relevant physiological process. For example, synthetic interaction between mutations in R1 and F2 can be deduced from the analytical analysis of the steady state by simultaneously setting $k_{-1} = 0$ and $k_2 = 0$, because it traps a toxic intermediate state. If we set k_{-1} and k_2 to very small values but not to zero, the steady state can correspond to a viable state but the kinetics of DNA repair can be so slow that in practice it will lead to cell death on the way to this steady state.

Simple analysis shows that it is not possible to set to zero the denominator *A* by placing to zero any single or a couple of k_is. Hence, this solution is applicable for all single and double mutants in the model. However, there exist triple mutants for which this solution should be modified (for example, if $k_{-1} = k_3 = k_1 = 0$ then *A*=0). We will not consider triple mutants in our analysis.

The general solution (3) includes one particular case when the compensatory pathway is much faster than the S → I → P cascade itself. Hence, DNA is repaired practically only through the compensatory pathway. In mathematical terms this corresponds to the case $k_3 >> k_1$. In our numerical simulations we will consider that the compensatory pathway is relatively slow, hence $k_1 >> k_3$. This will always be correct except for the F1↓ mutation ($k_1 \cong 0$), which should be treated separately. The requirement of a relatively slow compensatory pathway is not essential for most of our conclusions (see the parametric study in Figure 5). In particular, the kinetic

trapping mechanism due to inactivation of the second forward reaction (F2) and first backwards reaction (R1) (Figure 3) is always valid, although there could be a kinetic difference in the accumulation of the toxic intermediate.

Let us introduce notations for the relative speed of the compensatory pathway r_3, forward/backward rate ratios r_1 and r_2 for the reactions 1 and 2, and the ratio of backward rates r_b :

$$r_3 \equiv \frac{k_3}{k_1} << 1, \quad r_1 \equiv \frac{k_1}{k_{-1}}, \quad r_2 \equiv \frac{k_2}{k_{-2}}, \quad r_b \equiv \frac{k_{-1}}{k_{-2}}. \tag{4}$$

Then, from (3) we can formulate the steady-state cell viability conditions with respect to toxicity (*i.e.*, $I^s < [I]_{tox}$) and DNA damage ($S^s<[S]_{leth}$):

$$r_3 r_b + r_2 + \frac{1}{r_1} > \frac{1-[I]_{tox}}{[I]_{tox}}, \tag{5}$$

$$r_1\left[r_3 r_b + \left(1 + r_2\right)\right] > \frac{1-[S]_{leth}}{[S]_{leth}}, \tag{6}$$

From these conditions it follows qualitatively that for cell death from toxicity, three requirements should be satisfied simultaneously: $r_1 >> 1$ and $r_2 << 1$ and $r_b r_3 < \frac{1-[I]_{tox}}{[I]_{tox}}$.

For cell death from unrepaired DNA damage it is enough that $r_1 \ll \min\left(\frac{1}{r_b r_3}, \frac{1}{1-r_2}\right)$.

When the DNA repair pathway functions normally, we assume that both the S → I and I → P reactions are more efficient in the forward direction. In mathematical language, this is formulated as $r_1 >> 1$, $r_2 >> 1$.

B. Conditions for various cellular fates in the toy model

The five kinetic rates of the model can be grouped into three control parameters, r_1, r_2 and $r_3 \times r_b$, determining the cell viability (see (5) and (6)). Depending on whether they are large or small, the pathway can be found in one of the following states: Normal Robust (state **NR)**, Normal Fragile (state **NF)**, Compensated (state **C**), death due to DNA Damage (state **DD)** and Death due to Toxic intermediates (state **DT)** (Figure 4). The most sensitive parameter is the forward/backward rate ratio for the reaction I↔P, $r_2 \equiv \frac{k_2}{k_{-2}}$. From Figure 4 and Figure 5 it follows that the pathway can function in the normal mode only if r_2 is sufficiently large. For example, eliminating the forward reaction (putting $k_2 = 0$) makes this ratio equal to zero, which prevents the pathway from normal functioning and may produce accumulation of toxic intermediates or unresolved DNA damage.

The second most important parameter is a complex combination of kinetic rate parameters $r_3 \times r_b \equiv \frac{k_3}{k_1} \times \frac{k_{-1}}{k_{-2}}$. Since we assume that the compensatory pathway is relatively slow and $r_3 \equiv \frac{k_3}{k_1} << 1$ then the product $r_3 \times r_b$ can be large only if $r_b \equiv \frac{k_{-1}}{k_{-2}} >> \frac{1}{r_3} >> 1$. In the case of a large r_b, the cell has a possibility to be rescued by a compensatory pathway (state **C**, Figure 4).

The mathematical model predicts that the normal functioning of the pathway can proceed in two distinct modes: a more robust **NR** (large $r_3 \times r_b$) state or a more fragile **NF** state (small $r_3 \times r_b$). In

the normal fragile state **NF,** one single null mutation disrupting the pathway at the downstream I↔P step (single deletion of F2, Figure 6, row 8 and Figure S4 in Text S1) can lead to lethal consequences. In the normal robust state **NR,** the cell can resist to disruption of the I↔P step by utilizing the compensatory path and switching to the compensated state **C** (Figure 6, row 7 and Figure S4 in Text S1).

We define that if in the steady state it is more probable to find the system in the toxic intermediate state than in any other states then it causes cell death due to the toxic stress (state **DT**). If in the steady state it is more probable to find the systems in the unresolved DNA damage state then cell death is due to DNA damage (state **DD**). The forward/backward rate ratio $r_1 \equiv \frac{k_1}{k_{-1}}$ for the reaction S↔I plays a role in determining the cell fate between the death from unrepaired DNA damage (state **DD**) or from toxic amounts of DNA repair intermediates (state **DT**, Figure 4). In each context, there is a minimal rate r_1 needed for cell viability. The dependence of pathway states on the values of the control parameters is summarized in Figures 4 and 5.

Section S3

Normal pathway functioning does not require step reversibility. From the steady-state solution of the mathematical model it follows that the steady-state amount of repaired DNA [*P*] in the absence of the reverse reaction P → I ($k_{-2} = 0$) should be equal to 1.0, which means perfect DNA repair. If both forward reactions are much faster than the backward reactions ($k_1, k_2 >> k_{-1}, k_{-2}$) then the amount of unrepaired DNA is $1-[P] \approx \frac{k_{-2}}{k_2} \approx 0$, *i.e.* it does not depend on the backward rate constant of the reaction I → S. Hence, the efficiency of DNA repair depends very little on the rate of backward reactions unless they are much faster than the rates of the forward reactions. Moreover, theoretically the absence of reversibility of the reaction I → P causes no DNA repair defect.

Disruption of the DNA repair pathway at downstream step can lead to lethality from toxicity. From Figure 4 it follows that the normal functioning of the DNA repair pathway corresponds to the right column with both upper and bottom rows being possible. The bottom row corresponds to the situation when the backward rate of the first reaction S → I is much faster than the backward rate of the I → P reaction ($k_{-1}/k_{-2} >> 1$). Let us denote it as mode normal robust (NR). The upper row situation is implemented when these rates are comparable or the first is smaller than the second ($k_{-1} \le k_{-2}$). This is denoted as normal fragile (NF).

Single gene knock-out or over-expression can change one or two of the ratios r_1, r_2, r_3, r_b (for example, eliminating F1 affects both r_1 and r_3). Hence, it corresponds to the change of a cell shown in the Figure 4. As a result, changing cell fate from survival to death by changing the activity of a single enzyme is possible in both the NR and NF states by changing the r_2 ratio. This is possible by either disrupting the downstream forward step ($k_2 = 0$, knocking-out F2) or significantly increasing the backward rate ($k_{-2} >> k_2$, over-expression of R2). The latter is the only option for the NR state because knocking-out F2 in the NR state does not affect the $r_3 \times r_b$ product and leads only to the C state (in which DNA is only partially repaired, see Figure 6, row 7).

Certain synthetic gene interactions can be lethal. Changing the activities of a couple of enzymes can change the system state from NR or NF to the state C or the lethal states DD, DT. There are several possibilities. Notice that one of the mutations should necessarily affect the r_2 ratio from large to small, *i.e.* either knocking-down F2 or over-expressing R2. In the NF state this is already sufficient to provide lethal accumulation of toxic intermediate (DT state), see Figure 6, row 8. In the NR state, the second mutation should affect the $r_3 \times r_b$ product changing it from large to small. There are three possibilities: 1) knocking-down the compensatory pathway by removing EC, see Figure 6, row 4; 2) over-expressing F1, see Figure 6, row 13; 3) knocking-down R1 or overexpressing R2, see Figure 6, row 11.

Section S4

SBTOOLBOX description of the mathematical model used for numerical simulations in Matlab environment

```
********** MODEL NAME

Synthetic lethality in one pathway

********** MODEL STATES

d/dt(D) = -R1-R3

d/dt(I) = R1-R2

d/dt(R) = R2+R3

D(0) = 1

I(0) = 0

R(0) = 0

********** MODEL PARAMETERS

k1f = 10

k1b = 1

k2f = 5

k2b = 0.1

k3 = 2

********** MODEL VARIABLES
```

```
********** MODEL REACTIONS

R1 = k1f*D-k1b*I

R2 = k2f*I-k2b*R

R3 = k3*D

********** MODEL FUNCTIONS
```

Protein	Cancer type	Reference
BRCA1	Breast, ovarian, colon, prostate	[1,2]
BRCA2	Breast, prostate, ovarian, lung, bladder, cervical	[3-9]
RAD51	Breast, bladder, pancreatic	[10-13]
RAD51B	Breast	[14]
RAD51C	Breast, ovarian	[15-18]
RAD51D	Breast	[19]
XRCC2/XRCC3	Breast, lung, bladder, colorectal, ovarian, pancreatic, thyroid	[20-26]
RAD54	Pancreatic, colorectal, breast, cervical	[27-29]
RAD54B	Colon, lymphoma	[30]
BLM	Colorectal, lymphoma, bladder, breast, lung	[31-36]
FANCJ	Breast, bladder, lung	[37-39]
FANCM	Breast, pancreatic	[38,40]
RTEL1	Glioma	[41,42]
MUS81	Lymphoma, gastric, breast	[29,43-45]
EME1	Glioma	[46]
POLH	Breast, melanoma, bladder	[18,47,48]

Table S1. Involvement of HR proteins in cancer. Information extracted from Atlas of Genetics and Cytogenetics in Oncology and Haematology (http://atlasgeneticsoncology.org/), Genetic Association Database (http://geneticassociationdb.nih.gov), and the literature.

Supplemental References

1. Futreal PA, Liu Q, Shattuck-Eidens D, Cochran C, Harshman K, et al. (1994) BRCA1 mutations in primary breast and ovarian carcinomas. Science 266: 120-122.
2. Ford D, Easton DF, Bishop DT, Narod SA, Goldgar DE (1994) Risks of cancer in BRCA1-mutation carriers. Breast Cancer Linkage Consortium. Lancet 343: 692-695.
3. Mitra AV, Jameson C, Barbachano Y, Sodha N, Kote-Jarai Z, et al. (2010) Elevated expression of Ki-67 identifies aggressive prostate cancers but does not distinguish BRCA1 or BRCA2 mutation carriers. Oncology Reports 23: 299-305.
4. Kirova YM, Savignoni A, Sigal-Zafrani B, de La Rochefordiere A, Salmon RJ, et al. (2010) Is the breast-conserving treatment with radiotherapy appropriate in BRCA1/2 mutation carriers? Long-term results and review of the literature. Breast Cancer Research and Treatment 120: 119-126.
5. Kontorovich T, Levy A, Korostishevsky M, Nir U, Friedman E (2010) Single nucleotide polymorphisms in miRNA binding sites and miRNA genes as breast/ovarian cancer risk modifiers in Jewish high-risk women. International Journal of Cancer 127: 589-597.
6. Marsit CJ, Liu M, Nelson HH, Posner M, Suzuki M, et al. (2004) Inactivation of the Fanconi anemia/BRCA pathway in lung and oral cancers: implications for treatment and survival. Oncogene 23: 1000-1004.
7. Rebbeck TR, Mitra N, Domchek SM, Wan F, Chuai S, et al. (2009) Modification of ovarian cancer risk by BRCA1/2-interacting genes in a multicenter cohort of BRCA1/2 mutation carriers. Cancer Research 69: 5801-5810.
8. Neveling K, Kalb R, Florl AR, Herterich S, Friedl R, et al. (2007) Disruption of the FA/BRCA pathway in bladder cancer. Cytogenetic and Genome Research 118: 166-176.
9. Narayan G, Arias-Pulido H, Nandula SV, Basso K, Sugirtharaj DD, et al. (2004) Promoter hypermethylation of FANCF: disruption of Fanconi Anemia-BRCA pathway in cervical cancer. Cancer Research 64: 2994-2997.
10. Mathews LA, Cabarcas SM, Hurt EM, Zhang X, Jaffee EM, et al. (2011) Increased expression of DNA repair genes in invasive human pancreatic cancer cells. Pancreas 40: 730-739.
11. Johnson J, Healey S, Khanna KK, Chenevix-Trench G (2011) Mutation analysis of RAD51L1 (RAD51B/REC2) in multiple-case, non-BRCA1/2 breast cancer families. Breast Cancer Research and Treatment 129: 255-263.
12. Rodriguez-Lopez R, Osorio A, Ribas G, Pollan M, Sanchez-Pulido L, et al. (2004) The variant E233G of the RAD51D gene could be a low-penetrance allele in high-risk breast cancer families without BRCA1/2 mutations. International Journal of Cancer 110: 845-849.

13. Figueroa JD, Malats N, Rothman N, Real FX, Silverman D, et al. (2007) Evaluation of genetic variation in the double-strand break repair pathway and bladder cancer risk. Carcinogenesis 28: 1788-1793.
14. Figueroa JD, Garcia-Closas M, Humphreys M, Platte R, Hopper JL, et al. (2011) Associations of common variants at 1p11.2 and 14q24.1 (RAD51L1) with breast cancer risk and heterogeneity by tumor subtype: findings from the Breast Cancer Association Consortium. Human Molecular Genetics 20: 4693-4706.
15. Akbari MR, Tonin P, Foulkes WD, Ghadirian P, Tischkowitz M, et al. (2010) RAD51C germline mutations in breast and ovarian cancer patients. Breast Cancer Research 12: 404.
16. Zheng Y, Zhang J, Hope K, Niu Q, Huo D, et al. (2010) Screening RAD51C nucleotide alterations in patients with a family history of breast and ovarian cancer. Breast Cancer Research and Treatment 124: 857-861.
17. Monsees GM, Kraft P, Chanock SJ, Hunter DJ, Han J (2011) Comprehensive screen of genetic variation in DNA repair pathway genes and postmenopausal breast cancer risk. Breast Cancer Research and Treatment 125: 207-214.
18. Michiels S, Laplanche A, Boulet T, Dessen P, Guillonneau B, et al. (2009) Genetic polymorphisms in 85 DNA repair genes and bladder cancer risk. Carcinogenesis 30: 763-768.
19. Loveday C, Turnbull C, Ramsay E, Hughes D, Ruark E, et al. (2011) Germline mutations in RAD51D confer susceptibility to ovarian cancer. Nature Genetics 43: 879-882.
20. Silva SN, Tomar M, Paulo C, Gomes BC, Azevedo AP, et al. (2010) Breast cancer risk and common single nucleotide polymorphisms in homologous recombination DNA repair pathway genes XRCC2, XRCC3, NBS1 and RAD51. Cancer Epidemiology 34: 85-92.
21. Hung RJ, Christiani DC, Risch A, Popanda O, Haugen A, et al. (2008) International Lung Cancer Consortium: pooled analysis of sequence variants in DNA repair and cell cycle pathways. Cancer Epidemiology, Biomarkers & Prevention 17: 3081-3089.
22. Damaraju S, Murray D, Dufour J, Carandang D, Myrehaug S, et al. (2006) Association of DNA repair and steroid metabolism gene polymorphisms with clinical late toxicity in patients treated with conformal radiotherapy for prostate cancer. Clinical Cancer Research 12: 2545-2554.
23. Kweekel DM, Antonini NF, Nortier JW, Punt CJ, Gelderblom H, et al. (2009) Explorative study to identify novel candidate genes related to oxaliplatin efficacy and toxicity using a DNA repair array. British Journal of Cancer 101: 357-362.
24. Webb PM, Hopper JL, Newman B, Chen X, Kelemen L, et al. (2005) Double-strand break repair gene polymorphisms and risk of breast or ovarian cancer. Cancer Epidemiology, Biomarkers & Prevention 14: 319-323.
25. Enjuanes A, Benavente Y, Bosch F, Martin-Guerrero I, Colomer D, et al. (2008) Genetic variants in apoptosis and immunoregulation-related genes are associated with risk of chronic lymphocytic leukemia. Cancer Research 68: 10178-10186.
26. Garcia-Quispes WA, Perez-Machado G, Akdi A, Pastor S, Galofre P, et al. (2011) Association studies of OGG1, XRCC1, XRCC2 and XRCC3 polymorphisms with differentiated thyroid cancer. Mutation Research 709-710: 67-72.
27. McManus KJ, Barrett IJ, Nouhi Y, Hieter P (2009) Specific synthetic lethal killing of RAD54B-deficient human colorectal cancer cells by FEN1 silencing. Proceedings of the National Academy of Sciences USA 106: 3276-3281.

28. McWilliams RR, Bamlet WR, de Andrade M, Rider DN, Couch FJ, et al. (2009) Polymorphic variants in hereditary pancreatic cancer genes are not associated with pancreatic cancer risk. Cancer Epidemiology, Biomarkers & Prevention 18: 2549-2552.
29. Mavaddat N, Dunning AM, Ponder BA, Easton DF, Pharoah PD (2009) Common genetic variation in candidate genes and susceptibility to subtypes of breast cancer. Cancer Epidemiology, Biomarkers & Prevention 18: 255-259.
30. Hiramoto T, Nakanishi T, Sumiyoshi T, Fukuda T, Matsuura S, et al. (1999) Mutations of a novel human *RAD54* homologue, *RAD54B*, in primary cancer. Oncogene 18: 3422-3426.
31. Frank B, Hoffmeister M, Klopp N, Illig T, Chang-Claude J, et al. (2010) Colorectal cancer and polymorphisms in DNA repair genes WRN, RMI1 and BLM. Carcinogenesis 31: 442-445.
32. Schuetz JM, MaCarthur AC, Leach S, Lai AS, Gallagher RP, et al. (2009) Genetic variation in the NBS1, MRE11, RAD50 and BLM genes and susceptibility to non-Hodgkin lymphoma. BMC Medical Genetics 10: 117.
33. Guey LT, Garcia-Closas M, Murta-Nascimento C, Lloreta J, Palencia L, et al. (2010) Genetic susceptibility to distinct bladder cancer subphenotypes. European Urology 57: 283-292.
34. Hosgood HD, 3rd, Menashe I, Shen M, Yeager M, Yuenger J, et al. (2008) Pathway-based evaluation of 380 candidate genes and lung cancer susceptibility suggests the importance of the cell cycle pathway. Carcinogenesis 29: 1938-1943.
35. Ding SL, Yu JC, Chen ST, Hsu GC, Kuo SJ, et al. (2009) Genetic variants of BLM interact with RAD51 to increase breast cancer susceptibility. Carcinogenesis 30: 43-49.
36. Wirtenberger M, Frank B, Hemminki K, Klaes R, Schmutzler RK, et al. (2006) Interaction of Werner and Bloom syndrome genes with p53 in familial breast cancer. Carcinogenesis 27: 1655-1660.
37. McInerney NM, Miller N, Rowan A, Colleran G, Barclay E, et al. (2010) Evaluation of variants in the CHEK2, BRIP1 and PALB2 genes in an Irish breast cancer cohort. Breast Cancer Research and Treatment 121: 203-210.
38. Garcia MJ, Fernandez V, Osorio A, Barroso A, Fernandez F, et al. (2009) Mutational analysis of FANCL, FANCM and the recently identified FANCI suggests that among the 13 known Fanconi Anemia genes, only FANCD1/BRCA2 plays a major role in high-risk breast cancer predisposition. Carcinogenesis 30: 1898-1902.
39. Kote-Jarai Z, Jugurnauth S, Mulholland S, Leongamornlert DA, Guy M, et al. (2009) A recurrent truncating germline mutation in the BRIP1/FANCJ gene and susceptibility to prostate cancer. British Journal of Cancer 100: 426-430.
40. Barroso E, Pita G, Arias JI, Menendez P, Zamora P, et al. (2009) The Fanconi anemia family of genes and its correlation with breast cancer susceptibility and breast cancer features. Breast Cancer Research and Treatment 118: 655-660.
41. Wrensch M, Jenkins RB, Chang JS, Yeh RF, Xiao Y, et al. (2009) Variants in the CDKN2B and RTEL1 regions are associated with high-grade glioma susceptibility. Nature Genetics 41: 905-908.
42. Shete S, Hosking FJ, Robertson LB, Dobbins SE, Sanson M, et al. (2009) Genome-wide association study identifies five susceptibility loci for glioma. Nature Genetics 41: 899-904.
43. El Ghamrasni S, Pamidi A, Halaby MJ, Bohgaki M, Cardoso R, et al. (2011) Inactivation of chk2 and mus81 leads to impaired lymphocytes development, reduced genomic instability, and suppression of cancer. PLoS Genetics 7: e1001385.

44. Wu F, Shirahata A, Sakuraba K, Kitamura Y, Goto T, et al. (2010) Down-regulation of Mus81 as a potential marker for the malignancy of gastric cancer. Anticancer Research 30: 5011-5014.
45. Wu F, Shirahata A, Sakuraba K, Kitamura Y, Goto T, et al. (2011) Downregulation of Mus81 as a novel prognostic biomarker for patients with colorectal carcinoma. Cancer Science 102: 472-477.
46. Chang JS, Yeh RF, Wiencke JK, Wiemels JL, Smirnov I, et al. (2008) Pathway analysis of single-nucleotide polymorphisms potentially associated with glioblastoma multiforme susceptibility using random forests. Cancer Epidemiology, Biomarkers & Prevention 17: 1368-1373.
47. Hussain SK, Mu LN, Cai L, Chang SC, Park SL, et al. (2009) Genetic variation in immune regulation and DNA repair pathways and stomach cancer in China. Cancer Epidemiology, Biomarkers & Prevention 18: 2304-2309.
48. Di Lucca J, Guedj M, Lacapere JJ, Fargnoli MC, Bourillon A, et al. (2009) Variants of the xeroderma pigmentosum variant gene (POLH) are associated with melanoma risk. European Journal of Cancer 45: 3228-3236.
